# Baseline risk and marginal willingness to pay for health risk reduction

**DOI:** 10.1007/s11166-017-9267-x

**Published:** 2017-12-13

**Authors:** Shelby Gerking, Wiktor Adamowicz, Mark Dickie, Marcella Veronesi

**Affiliations:** 1 Department of Economics and Tilburg Sustainability Center, Tilburg University, P.O. Box 90153, 5000 LE Tilburg, The Netherlands; 2 Department of Resource Economics and Environmental Sociology, University of Alberta, Edmonton, Alberta T6G 2H1 Canada; 3 Department of Economics, University of Central Florida, P.O. Box 1400, Orlando, FL 32816-1400 USA; 4 Department of Economics, University of Verona, Via Cantarane 24, 37129 Verona, Italy; 5 Center for Development and Cooperation, ETH Zurich, Clausiusstrasse 37, 8092 Zurich, Switzerland

**Keywords:** Baseline risk, Morbidity, Willingness to pay, Heart disease, Health risk, Endogenous risk, Children’s morbidity risk, D61, I12, I38, J13, Q51, Q58

## Abstract

Empirical results presented in this paper suggest that parents’ marginal willingness to pay (MWTP) for a reduction in morbidity risk from heart disease is inversely related to baseline risk (i.e., the amount of risk initially faced) both for themselves and for their children. For instance, a 40% reduction from the mean of baseline risk results in an increase in MWTP by 70% or more. Thus, estimates of monetary benefits of public programs to reduce heart disease risk would be understated if the standard practice is followed of evaluating MWTP at initial risk levels and then multiplying this value by the number of cases avoided. Estimates are supported by: (1) unique quantitative information on perceptions of the risk of getting heart disease that allow baseline risk to be defined at an individual level and (2) improved econometric procedures to control for well-known difficulties associated with stated preference data.

**Electronic supplementary material:**

The online version of this article (10.1007/s11166-017-9267-x) contains supplementary material, which is available to authorized users.

## Introduction

Estimation of monetary benefits of reductions in risk to human health is central to analysis of policies affecting the environment, transportation, and workplace safety because: (1) protection of human health is emphasized in both the statutory and regulatory framework of the U.S. and other countries and (2) benefit-cost analysis is commonly used as an evaluation tool when considering policy changes. As illustrated in recent analyses of more stringent diesel fuel standards (U.S. Environmental Protection Agency 2000) and air quality regulations (U.S. Environmental Protection Agency 2011), estimates of total health benefits generally are obtained by multiplying a marginal willingness to pay (MWTP) measure by the number of illnesses or deaths avoided. This approach rests on the assumption that the MWTP for health risk reduction is independent of baseline risk (i.e., the amount of risk initially faced). If MWTP for health risk reduction varies with baseline risk, however, accuracy of total health benefit estimates in these and other policy evaluations may be compromised.

Prior empirical studies provide a weak basis for making more refined calculations of total health benefits in light of their conflicting evidence on the relationship between MWTP for health risk reduction and baseline risk. Smith and Desvousges (1987), Lillard and Weiss (1997), Viscusi and Aldy (2003), and Edwards (2008), for instance, find evidence indicating that MWTP to reduce health risk declines as baseline risk rises, but results in Viscusi and Evans (1990), Sloan et al. (1998), and Finkelstein et al. (2013) support the opposite relationship*.* Hammitt and Haninger (2010) and Alberini and Scasny (2013) find that MWTP for a unit of health risk reduction is insensitive to changes in baseline risk.

This paper examines the relationship between MWTP to reduce an aspect of health risk—morbidity risk from heart disease—and baseline risk of this disease. This focus is important for four reasons. First, heart disease is the leading cause of death globally (World Health Organization 2014) and for most ethnicities in the U.S. (Heron 2012). Second, benefits of reduced morbidity from heart disease as well as from many other illnesses have been understudied relative to benefits of reduced mortality.^1^ Third, morbidity risk from heart disease can be viewed as a component of mortality risk (mortality risk is equal to morbidity risk multiplied by the conditional risk of death given illness); therefore, evidence found for morbidity on the relationship between MWTP and baseline risk may provide insights into the corresponding relationship for mortality. Fourth, heart disease risk may be influenced by individual behavior and thus provides a natural basis for examining the relationship between MWTP and baseline risk when risk may be endogenous (i.e., influenced by agent behavior).

An expected utility model provides the foundation to support and to more fully interpret empirically estimated relationships between MWTP to reduce heart disease risk and baseline risk. The model is framed in terms of a risk that either can be exogenous or endogenous. As demonstrated later on, the direction of change in MWTP to reduce risk with changes in baseline risk depends on whether risk is fixed or subject to agent control together with the health state dependence of the marginal utility of consumption, an issue that is central to treatments of optimal insurance, life-cycle savings, and portfolio choice (Edwards 2008; Finkelstein et al. 2009, 2013).

The model also features a parent that makes decisions for herself and one child. This characterization of the household permits development of an optimality condition (indicating how much parents are willing to pay to protect themselves from heart disease compared to how much they are willing to pay to protect their children) that necessarily holds if risk is endogenous, but does not necessarily hold if risk is exogenous. Further advantages of distinguishing parents from children in the analysis are: (1) children often are singled out for special treatment in policy analyses (Clinton 1997), (2) household MWTP to reduce health risks to children may differ from household willingness to pay to reduce similar risks to adults.^2^

Data utilized in the empirical analysis are drawn from a nationally representative stated preference survey conducted in the U.S. on the risk of getting heart disease. Four features of these data and the empirical methods are particularly important. First, an advantage of the data is that they include unique quantitative information on perceptions of the risk of getting heart disease that allow baseline risk to be defined at an individual level both for parents and their children. This approach avoids the concern expressed by Viscusi (2012, p. 764) that respondents in prior stated preference studies may not believe they face the levels of baseline risk assigned to them. Second, stated preference estimates of MWTP are obtained based on parents’ purchase intentions for a hypothetical good (a vaccine) that lowers heart disease risk. Thus, the survey incorporates the idea that risk is subject to parent control, however, it is designed to avoid cues that induce parents to express particular purchase intentions. Moreover, parents are unlikely to be aware of the abovementioned optimality condition in the endogenous risk model as they complete the survey. Third, improved econometric procedures are developed to control for well-known difficulties associated with stated preference data including the problem that people may not accurately report their intentions to purchase a hypothetical good when they do not actually have to pay for it. Fourth, alternative functional forms of the relationship between MWTP to reduce risk and baseline risk are investigated.

The main empirical finding suggests that parents’ MWTP for a reduction in heart disease risk increases as baseline risk declines both for themselves and for their children. For instance, a 40% reduction from the mean of baseline heart disease risk results in an increase in MWTP by 70% or more for both parents and children. Taken by itself, this finding could be consistent with either the exogenous risk or endogenous risk formulation of the conceptual model, but in either case calls into question the conventional practice in policy analysis of obtaining total health benefit estimates by multiplying an estimate of MWTP for health risk reduction by the number of cases avoided. Additionally, the inclusion of children in the analysis yields two further insights. First, estimates suggest that parents are willing to pay the same amount at the margin for given absolute or proportionate risk reductions in heart disease risk for themselves and for their children, an outcome that is consistent with the endogenous risk model. Second, this finding, together with the inverse relationship found between MWTP for heart disease risk reduction and baseline risk of this disease, is consistent with the notion that the marginal utility of income may be greater in the healthy state than in the sick state.

## Conceptual framework

This section makes use of one-period, two-person × two-state expected utility models of a parent (*p*) that makes decisions for herself and one child (*k*). The models: (1) contrast the determination of MWTP to reduce morbidity risk and its relationship to baseline risk when risk is treated as exogenous or as endogenous (i.e., influenced by parent behavior) and (2) represent simple extensions of work by Pratt and Zeckhauser (1996) and Liu and Neilson (2005, 2006). Of course, more complex models could be developed featuring two parents and/or more than one child. Such models, for example, might consider cooperative vs. non-cooperative behavior of parents together with the possibility that parents may treat children unequally. These complications for the most part are set aside in this section, although an extension of the analysis to the case of one parent with more than one child is briefly discussed. The main purpose of this section is to focus on relationships between baseline risk and MWTP to reduce risk that can be used to guide and interpret the empirical work presented in Sections 4 and 5.

### Exogenous risk

In the case where morbidity risk is exogenously determined, the parent’s *ex ante* expected utility is given by the probability weighted sum of her utilities in each of four health states in which the parent and child are either healthy (*H*) or sick (*S*).^3^ The illness might be thought of as heart disease (the subject of empirical analysis in subsequent sections of the paper) or another non-communicable disease that may keep people in less than perfect health for an extended period and may lead to eventual death.^4^ The parent’s expected utility (*EU*) is

1 $$ EU=\left(1-{R}_p\right)\left(1-{R}_k\right){U}_{HH}+{R}_p\left(1-{R}_k\right){U}_{SH}+{R}_k\left(1-{R}_p\right){U}_{HS}+{R}_p{R}_k{U}_{SS} $$

In Eq. (1), *U*_*ij*_ refers to the parent’s utility in health state *ij* where *i* indicates the parent’s health state (*i* = *H*, *S*) and *j* denotes the child’s health state (*j* = *H*, *S*) (for instance, *U*_*SH*_ denotes the parent’s utility if she is sick and the child is healthy), and *R*_*p*_ and *R*_*k*_ denote probabilities that the parent and child are sick possibly as a result of exposure to toxins at home, at work, or while commuting.^5^ Utility in all health states increases at a non-increasing rate with the size of an exogenous income endowment (*I*); thus *U*_*ij*_ = *U*_*ij*_(*I*) and ${U}_{ij}^{\prime }(I)>0,\kern0.5em {U}_{ij}^{{\prime\prime} }(I)\le 0$ where the prime superscripts denote differentiation. Income may differ between health states, but these differences are subsumed in the four health state utility functions.

Equation (1) can be simplified by focusing attention on the parent.^6^

2 $$ EU=\left(1-{R}_p\right){EU}_{H\cdotp }+{R}_p{EU}_{S\cdotp } $$

In Eq. (2), *EU*_*H*·_ = (1 − *R*_*k*_)*U*_*HH*_ + *R*_*k*_ *U*_*HS*_ denotes expected utility of the parent if healthy given that the child is either healthy or sick and *EU*_*S*·_ = (1 − *R*_*k*_)*U*_*SH*_ + *R*_*k*_ *U*_*SS*_ denotes expected utility of the parent if sick given that the child is either healthy or sick. Expected utility if sick could be positive, however *EU*_*H*·_ is assumed to be greater than *EU*_*S*·_ (*EU*_*H*·_ − *EU*_*S*·_ > 0) at all levels of income.

For a given morbidity risk to the child, the parent’s MWTP to reduce her own morbidity risk (*MWTP*_*p*_) by a small amount at unchanged expected utility is

3 $$ {MWTP}_p=\frac{\partial I}{\partial {R}_p}=\frac{\left({EU}_{H\cdotp }-{EU}_{S\cdotp}\right)}{\left(1-{R}_p\right)E{U}_{H\cdotp}^{\prime }+{R}_pE{U}_{S\cdotp}^{\prime }}>0 $$

In Eq. (3), the numerator of the ratio on the right hand side is interpreted as the parent’s expected utility gain when she is healthy rather than sick, given that the child may either be healthy or sick, and the denominator is interpreted as the marginal expected utility of income (assumed to be positive at all levels of income). Thus, the parent’s MWTP to reduce her own risk is positive (*MWTP*_*p*_ > 0).

Does the parent’s MWTP to reduce morbidity risk increase or decrease with an increase in baseline risk? If the marginal expected utility of income is larger when the parent is healthy than when sick, *MWTP*_*p*_ increases with an increase in *R*_*p*_ (i.e., *∂* ^2^ *I*/*∂R*_*p*_ *∂R*_*p*_ > 0). This outcome might be referred to as the “sick anyway” effect because higher values of *R*_*p*_ (representing higher values of baseline risk) boost the probability that the payment would come from the sick health state *S* in which the parent has a smaller marginal utility of income. The “sick anyway” effect is directly analogous to the “dead anyway” effect described in detail by Pratt and Zeckhauser (1996). Conversely, if the parent’s marginal expected utility of income is smaller when healthy than when sick, the value of *MWTP*_*p*_ decreases with an increase in baseline risk (*∂* ^2^ *I*/*∂R*_*p*_ *∂R*_*p*_ < 0). Viscusi and Evans (1990) and Finkelstein et al. (2009) demonstrate that the effect of illness on the marginal utility of income is both theoretically and empirically ambiguous; thus either a positive or negative relationship between baseline risk and MWTP to reduce morbidity risk is possible.

Corresponding results establish that for a given morbidity risk to herself, the parent’s MWTP to reduce morbidity risk to the child is

4 $$ {MWTP}_k=\frac{\partial I}{\partial {R}_k}=\frac{\left({EU}_{\cdotp H}-{EU}_{\cdotp S}\right)}{\left(1-{R}_k\right)E{U}_{\cdotp H}^{\prime }+{R}_kE{U}_{\cdotp S}^{\prime }}>0 $$

Notation and discussion pertaining to Eq. (4) parallels that for Eq. (3). The parent’s MWTP to reduce her own morbidity risk may be greater than, equal to, or less than her MWTP to reduce her child’s morbidity risk. In Eqs. (3) and (4), the expressions for the marginal expected utility of income are equivalent, but the relative magnitudes of (*EU*_*H*·_ − *EU*_*S*·_) and (*EU*_·*H*_ − *EU*_·*S*_) are unknown.

### Endogenous risk

The expected utility model just considered can be reformulated to make health risk endogenous (as in Liu and Neilson 2005, 2006). If attention is focused on the parent, expected utility can be expressed as

5 $$ EU=\left(1-{R}_p\left({X}_p,G\right)\right){EU}_{H\cdotp }+{R}_p\left({X}_p,G\right){EU}_{S\cdotp } $$

In Eq. (5), *EU*_*H*·_ = (1 − *R*_*k*_(*X*_*k*_, *G*))*U*_*HH*_ + *R*_*k*_(*X*_*k*_, *G*)*U*_*HS*_ denotes expected utility of the parent if healthy given that the child is either healthy or sick and *EU*_*S*·_ = (1 − *R*_*k*_(*X*_*k*_, *G*))*U*_*SH*_ + *R*_*k*_(*X*_*k*_, *G*)*U*_*SS*_ denotes expected utility of the parent if sick given that the child is either healthy or sick. Additionally, *X* = *X*_*p*_ + *X*_*k*_ denotes household (private) expenditures on health risk reduction for the benefit of the parent or child (assumed to be ubiquitously available at unit cost), and *G* represents expenditures on health risk reduction (perhaps by the public sector) that are exogenous to the household.

Utility in all health states is a function of consumption, defined as the exogenous income endowment net of the amount spent on private risk-reducing efforts (*I* − *X*); thus, *U*_*ij*_ = *U*_*ij*_(*I* − *X*), and *U* ^′^(*I* − *X*) > 0,  *U* ^″^(*I* − *X*) ≤ 0. Expenditures on *X* and *G* reduce the parent’s and child’s probability of illness and exhibit diminishing marginal productivity (i.e., ${R}_i^X\left({X}_i,G\right)<0,\kern0.5em {R}_i^{XX}\left({X}_i,G\right)>0$, ${R}_i^G\left({X}_i,G\right)<0,\kern0.5em {R}_i^{GG}\left({X}_i,G\right)>0$ (*i* = *p*, *k*)). Additionally, ${R}_i^{XG}\left({X}_i,G\right)$ may be either positive or negative depending on the nature of the expenditure, although Liu and Neilson (2006, pp. 2066, 2071) argue that in many settings, an increment in *G* makes *X* less productive in reducing risk (i.e., ${R}_i^{XG}\left({X}_i,G\right)>0$).^7^

The parent’s MWTP for a small reduction in morbidity risk for herself *R*_*p*_ can be found from first order conditions of the model. The first order condition for *X*_*p*_ is

6 $$ {R}_p^x\left({X}_p,G\right)\left({EU}_{S\cdotp }-{EU}_{H\cdotp}\right)-\left(1-{R}_p\left({X}_p,G\right)\right)E{U}_{H\cdotp}^{\prime }-{R}_p\left({X}_p,G\right)E{U}_{S\cdotp}^{\prime }=0 $$

Rearranging terms and multiplying by −1 yields

7 $$ {MWTP}_p^{\ast }=\frac{EU_{H\cdotp }-{EU}_{S\cdotp }}{\left(1-{R}_p\left({X}_p,G\right)\right)E{U}_{H\cdotp}^{\prime }+{R}_p\left({X}_p,G\right)E{U}_{S\cdotp}^{\prime }}=-\frac{1}{R_p^X\left({X}_p,G\right)} $$

Equation (7) shows that the parent’s MWTP to reduce her own morbidity risk in the endogenous risk model (${MWTP}_p^{\ast }$) equates to the marginal cost of risk reduction. Thus, in this model, private expenditures to reduce morbidity risk are undertaken up to the point where MWTP equates to marginal cost (see Harrington and Portney 1987 and Liu and Neilson 2006 for further discussion). Correspondingly, first order conditions for *X*_*k*_ show that in the endogenous risk model, the parent’s MWTP to reduce morbidity risk to the child equates to her marginal cost of risk reduction.

8 $$ {MWTP}_k^{\ast }=-\frac{1}{R_k^X\left({X}_k,G\right)} $$

### Comparison of model implications

Solutions for ${MWTP}_p^{\ast }$ and ${MWTP}_k^{\ast }$ (Eqs. (7) and (8)) differ from their counterparts for the exogenous risk case (Eqs. (3) and (4)) in at least six ways that are relevant to the empirical analysis presented in Sections 4 and 5. First, in obtaining MWTP for morbidity risk reduction in the endogenous risk model, utility terms are eliminated by accounting for optimization of private risk reducing expenditures.^8^ This outcome is not a feature of the exogenous risk model.

Second, MWTP for morbidity risk reduction can be positively or negatively related to changes in baseline risk in both the exogenous and endogenous risk models although the reasoning leading to this conclusion is somewhat different in each case. In the exogenous risk model, as previously noted, this relationship depends on whether the marginal expected utility of income when healthy is greater or less than the marginal expected utility of income when sick. In the endogenous risk model, on the other hand, health state dependence of the marginal utility of consumption plays a role, but the ambiguous sign of ${R}_i^{XG}$ also must be taken into consideration. This outcome can be seen by differentiating *MWTP* ^∗^ with respect to *G* as shown in Eq. (9) and noting that a larger value of *G* corresponds to a lower level of health risk.

9 $$ \frac{dMWTP_i^{\ast }}{dG}=\frac{\left({R}_i^{XX}\left({dX}_i/ dG\right)+{R}_i^{XG}\right)}{{\left({R}_i^X\right)}^2} $$

The numerator on the right hand side of Eq. (9) is composed of two terms: (1) ${R}_i^{XG}$ represents the direct effect of an increase in *G* on the productivity of *X*_*i*_ in reducing *R*_*i*_ and (2) ${R}_i^{XX}\left({dX}_i/ dG\right)$ represents the indirect effect that occurs through re-optimization of the choice of *X*_*i*_.

Consider the case in which an increase in *G* reduces the productivity of *X* in reducing risk $\left({R}_i^{XG}>0\right)$ and the marginal expected utility of consumption is larger when healthy rather than when sick. In this situation, ${R}_i^{XX}\left({dX}_i/ dG\right)<0$ (i.e., there is offsetting behavior), but the direct effect outweighs the re-optimization effect provided that an additional technical restriction is satisfied (see Liu and Neilson 2006, pp. 2068–2069). Thus, *∂MWTP* ^∗^/*∂G* > 0 and because ${R}_i^G<0$ MWTP for risk reduction is negatively related to baseline risk. This result, which is opposite to the “sick anyway” effect in the exogenous risk model, would be reversed if ${R}_i^{XG}<0$ and the parent’s marginal expected utility of consumption is smaller when the individual is healthy rather than sick.

Third, the endogenous risk model restricts the relative magnitudes of the parent’s marginal values of risk reduction between family members via an optimality condition; in contrast, the exogenous risk model imposes no corresponding requirement. In the endogenous risk model, first order conditions require that

10 $$ \frac{MWTP_p^{\ast }}{MWTP_k^{\ast }}=\frac{R_k^X\left({X}_k,G\right)}{R_p^X\left({X}_p,G\right)} $$

Consider the case in which one unit changes in *X*_*p*_ and *X*_*k*_ result in equal absolute reductions in morbidity risk for the parent and the child: ${R}_p^X\left({X}_p,G\right)={R}_k^X\left({X}_k,G\right)$. In this situation, the marginal cost of achieving each of the two risk reductions is the same because the price of *X* is the same for the parent and the child. Thus, the parent is willing to pay an equal amount of money at the margin for equal reductions in morbidity risk to each person. A similar argument establishes that if one unit changes in *X*_*p*_ and *X*_*k*_ result in equal *proportionate* morbidity risk reductions for parent and child, then the parent equates MWTP for equal proportionate risk reductions for herself and for her child. In this case, however, the parent’s MWTP *per unit* of morbidity risk reduction for herself and for her child will depend on the initial level of risk (i.e., baseline risk) faced by each as shown in Eq. (11)

11 $$ \frac{MWTP_p^{\ast }}{MWTP_k^{\ast }}=\frac{R_k}{R_p} $$

Fourth, the distinction between absolute and proportionate risk reductions in the endogenous risk model is important for interpreting the survey design and empirical methods discussed subsequently. As shown in Eqs. (7) and (8) ${MWTP}_i^{\ast }$ depends on the level of risk, which itself depends on parameters of the production function and the levels of *X*_*i*_ and *G*. The endogenous input *X*_*i*_ in turn depends on its price, income and the level of *G* (see Eq. (9)). In other words, the absolute risk reduction obtained from an additional unit of *X*_*i*_ (${R}_i^X$) is in general endogenous. In the special case in which *X*_*i*_ is a perfect substitute for *G*, however, Mäler’s (1974, pp. 178–180) analysis implies that the risk reduction associated with an additional unit of *X*_*i*_ is exogenous and ${MWTP}_i^{\ast }$ equates to the constant marginal cost of risk reduction. As discussed in Section 3, respondents in the survey made decisions about risk-reducing goods that provided randomly assigned proportionate risk reductions independently of individual characteristics or behaviors. Thus the *X*_*i*_ goods considered in the empirical analysis are perfect substitutes for other inputs in the production of proportionate risk reductions, and the proportionate risk reductions are exogenous.

Fifth, suppose that the endogenous risk model is extended to consider the behavior of a parent with multiple children. In this case, a parent’s MWTP to reduce morbidity risk to herself or to any one of her children still would equate to marginal cost. Parents with more children, therefore, would find it more costly to reduce health risk for the entire family, however, the number of children present in a family may not alter the marginal evaluation of risk reduction for an individual child.^9^ For instance, if one unit changes in the use of *X* result in equal proportionate risk reductions, then Eq. (11) would hold for the parent and any one of the children. If a further restriction is imposed that all children are identical, then the parent’s MWTP per unit of risk reduction would be equal for each of the children because each child would initially face the same amount of risk. Children within a family of course are unlikely to be identical, may differ along any number of dimensions (e.g., gender, age, birth order, health status) and therefore would be expected to face different amounts of risk. In this situation, a parent’s ratio of MWTP values to reduce risk to any two children would depend on these differing amounts of risk similarly to Eq. (11).

Sixth, a further distinction between the endogenous and exogenous risk models may be obtained by examining the relationship between MWTP for risk reduction and income. For instance, suppose that MWTP is inversely related to baseline risk. In this situation, the endogenous risk model predicts that an income increase would lead to a higher level of *X*_*i*_, a lower level of *R*_*i*_, and a higher marginal cost of risk reduction. Thus, MWTP to reduce morbidity risk increases with income because MWTP equates to marginal cost. The exogenous risk model also predicts that MWTP to reduce morbidity risk increases with income. Nonetheless, in this model morbidity risk is fixed as income changes, whereas in the endogenous risk case the amount of *G* is fixed and *R*_*p*_ responds to a change in *X*_*p*_ brought about by a change in income.

## Data

Data are drawn from a survey of heart disease risks administered in 2011 to members of Knowledge Networks, Inc.’s national online research panel.^10^ The survey instrument reflected information obtained from: (1) two focus groups of 12 parents each conducted in a hotel conference room in Orlando, Florida and (2) an online pretest using 505 parents from the Knowledge Networks panel. The terms “coronary artery disease” and “heart disease” were used interchangeably in the survey. Data obtained are fully described in Dickie and Gerking (2011).

Knowledge Networks, Inc. panel members were eligible to participate in the study if they were parents aged 18 to 55 years, with at least one biological child aged 6 to 16 years living in the home, and without a prior diagnosis of heart disease. Parents with these characteristics were identified by sending a brief questionnaire to 7135 panel members. Of these panel members, 4309 (60.4%) completed the questionnaire and, of those, 3155 (73%) had the desired characteristics and completed at least a portion of the survey.^11^ Parents with a prior history of coronary artery disease were excluded to focus on *ex ante* perception and valuation of risk. Older teenagers were excluded because they are more likely than younger children to earn income and make independent consumption decisions. Children under age 6 years were excluded because in focus groups parents expressed difficulty in assessing and valuing their heart disease risk.

The total sample was divided into three subsamples: (1) 505 parents included in the pretest of the survey instrument, (2) 434 matched pairs of spouses (868 parents) living together in the same household, and (3) 1782 parents living in different households. This study is based on data from the subsample of unmatched parents plus the first randomly recruited parent for each of the matched pairs. One observation from the matched parents subsample and four observations from the unmatched parents subsample were excluded because respondents failed to complete the entire survey, so the number of observations available is *n* = 2211.^12^ Parents included in the pretest are excluded because the final version of the survey instrument differed from the pretest version. The second of the parents recruited for each of the matched pairs also were excluded for two reasons: (1) to avoid the possible contamination that may result if answers to survey questions were discussed between spouses and (2) because the matched pairs subsample is not exactly congruent with the subsample of parents living in different households. In the latter subsample, parents are queried about a child living in their household, whereas in the matched pairs subsample, the two parents are queried about the same child.

For the 74% of parents with two or more children living at home, one child was randomly selected and designated as the sample child. The decision to focus the empirical analysis on a single child is consistent with the conceptual framework in Section 2, but it also kept the survey to a manageable length and avoided burdening parents with repetitive questioning about multiple children. Roughly half (51.4%) of the sample children were male and the average age of sample children was 11 years.

### Initial risk perceptions

Parents provided estimates of risk of being diagnosed with coronary artery disease before age 75 years using a computer-based interactive grid scale similar to those used by Corso et al. (2001), and Viscusi and Huber (2012). As shown in the electronic supplementary material (Fig. A1), the grid depicted 100 numbered squares arranged in 10 rows and 10 columns that initially were colored blue. Parents used a computer mouse to re-color squares from blue to red to represent risk levels. Parents completed a tutorial about measuring risk in “chances in 100” before using the scale to estimate the risk. First, they were shown examples of scales representing various risk levels and were told how these scales represented “chances in 100.” Second, parents used the risk scales to represent the chances of experiencing an auto accident for each of two hypothetical people; Mr. A, a particularly bad driver that had 33 chances in 100 of an accident and Ms. B, a more careful driver that had 1 chance in 100 of an accident. Respondents then were asked which of these two people had the lesser chance of an accident. The 11% of parents that answered incorrectly were provided with additional review of the risk scales and then correctly identified the individual with lower risk. All respondents, regardless of their performance on the auto accident question, were retained in the empirical analysis.^13^ After completing the tutorial, they used the risk scale to estimate chances in 100 of getting coronary artery disease before age 75, first for themselves and then for the sample child.

Parents’ initial assessments of heart disease risks for themselves and for their child are summarized in Table 1. Important features of these subjective risk perceptions are: (1) fathers’ perceptions of heart disease risk on average exceeded those for mothers by 2.15 percentage points; the null hypothesis of no difference between mean risk perceptions of fathers and mean risk perceptions of mothers is rejected at the 5% level (but not at the 1% level) in an independent samples test, (2) parents perceived that their own risk of heart disease significantly exceeded the risk faced by their children; the null hypotheses that mean risk assessments are equal for mothers and their children and for fathers and their children are rejected at the 1% level in matched samples tests, (3) average parental assessments of children’s risks closely match epidemiological estimates, and (4) the average mother appears to have overestimated her risk, whereas the average father’s assessment is relatively close to epidemiological estimates of this risk for men.^14^

**Table 1:** Relative Frequency Distribution of Parents’ Assessments of Risk of Coronary Artery Disease Diagnosis before Age 75 (*n* = 2211)^a^

Chances in 100		Mothers’ Risk Estimates for (*n* = 1461)				Fathers’ Risk Estimates for (*n* = 750)			
		Self		Child		Self		Child	
From	Through	Initial	Revised	Initial	Revised	Initial	Revised	Initial	Revised
0	9	0.097	0.055	0.139	0.085	0.085	0.052	0.140	0.089
10	19	0.141	0.140	0.187	0.246	0.128	0.117	0.213	0.297
20	29	0.216	0.314	0.223	0.336	0.227	0.289	0.248	0.372
30	39	0.124	0.174	0.120	0.133	0.101	0.192	0.120	0.104
40	49	0.070	0.082	0.079	0.055	0.075	0.091	0.072	0.043
50	59	0.183	0.118	0.164	0.082	0.177	0.119	0.148	0.072
60	69	0.075	0.048	0.040	0.014	0.087	0.069	0.025	0.009
70	79	0.062	0.048	0.028	0.010	0.068	0.043	0.016	0.005
80	89	0.021	0.011	0.012	0.005	0.035	0.025	0.012	0.007
90	100	0.013	0.009	0.008	0.005	0.017	0.013	0.005	0.001
	Mean	35.23	32.69	29.45	24.98	37.38	35.34	27.16	22.95
Standard Deviation		22.18	19.01	20.20	15.40	23.16	20.14	18.98	14.23

^a^For all parents, regardless of gender, the mean of own initial perceived risk is 35.96, the mean of own revised perceived risk is 33.59, the mean of their children’s initial perceived risk is 28.67, and the mean of their children’s revised perceived risk is 24.30

### Revised risk assessments

Parents had the opportunity to revise their initial risk estimates after considering information about heart disease. Parents were told that the average person has about 27 chances in 100 of being diagnosed with coronary artery disease before age 75. This average risk was illustrated using a risk scale showing the 27% risk level next to the risk scales that parents had marked for themselves and their children. Parents then were told that they and their children probably would not have the same risk as the average person, because chances of getting heart disease depend on six risk factors that are different for everyone: gender, smoking, current health status, family history, exercise, and diet. The survey elicited information from parents about each of these risk factors while also providing information about how the factors influence risk. For example, parents’ smoking status was assessed and respondents were advised that coronary artery disease risks are higher for the average smoker than for the average non-smoker. Risk levels for smokers and non-smokers were illustrated using risk scales.

After reviewing the risk factors, parents were shown their initial risk assessments using the risk scales and were allowed to revise their assessments if desired. Revised risk assessments are shown in Table 1. Parents revised their own risk assessments about as frequently as they revised assessments of their children’s risk. About 47% of parents made revisions. Downward revisions predominated. Parents on average reduced their own risk assessment by two to three percentage points and reduced their assessment for their children by about four points. In separate matched samples tests, each of the hypotheses that (1) mean revised assessments are equal for mothers and their children and (2) mean revised assessments are equal for fathers and their children is rejected at the 1% level.

### Willingness to pay

The final section of the survey elicited stated preferences for hypothetical vaccines to reduce risk of coronary artery disease. The vaccines are treated as newly available private goods (like the good *X* in the endogenous risk model) and are introduced to operationalize Eqs. (7) and (8) in Section 2. They also: (1) yield no direct utility to the parent and (2) provide an incremental reduction in the amount of heart disease risk protection that would have been chosen in its absence. Parents’ purchase intentions were elicited for each of two vaccines.^15^ One of the vaccines reduced risk for the parent and the other vaccine reduced risk for the child. The two vaccines were presented one at a time in random order.^16^ Parents were told that the vaccines would slow the build-up of fatty deposits in the arteries, would be taken by injection annually, and would provide additional protection from coronary artery disease beyond the benefits that could be obtained from eating right and getting enough exercise.

Two characteristics of the vaccines, marginal product of risk reduction (i.e., effectiveness) and price, were varied randomly across respondents. Proportionate risk reductions of either Δ_*p*_ = 0.1 or Δ_*p*_ = 0.7 were assigned to parents and proportionate risk reductions of either Δ_*k*_ = 0.2 or Δ_*k*_ = 0.8 were assigned to their children. Thus, marginal products of the vaccines in reducing risk can be expressed as Δ_*i*_ *R*_*i*_, where *R*_*i*_ now denotes parents’ revised perceived risk for themselves and for their children. Proportions were assigned so that the value for the child always exceeded the value for the parent. Parents were told that risk reductions: (1) would materialize only for those who continued to receive the vaccinations annually through age 75 and (2) are larger for children because the vaccination program would be initiated earlier in life.

Vaccine prices assigned to parents were randomly drawn from among five values ($ *Q* = $10, $20, $40, $80, $160) selected on the basis of focus group input and pretest results. Prices varied only across households: the prices of the vaccines presented to parents for their own vaccine and for their child’s vaccine were the same. The idea behind the willingness to pay questions was to ask each parent if she would pay $ *Q* for a risk reduction of Δ_*k*_ *R*_*k*_ for the child and $ *Q* for a risk reduction of Δ_*p*_ *R*_*p*_ for herself.

The purchase scenario was introduced by: (1) reminding parents about symptoms of heart disease and the need for medical treatment and (2) presenting individualized hazard functions for themselves and their children to show how cumulative heart disease risk would be expected to increase between the present and age 75. Each parent then read the description of each vaccine and examined new risk scales for herself and for her child that showed: (1) the marginal product of the vaccine in reducing risk calculated on the basis of parent revised risk estimates (i.e., the number of risk squares eliminated, Δ_*i*_ *R*_*i*_) and (2) the amount of (revised) risk that would remain if the vaccine was purchased. After examining the new risk scales, parents were provided a budget constraint reminder, told that the vaccination program would continue in future years, and were asked about their intentions to purchase the vaccines for themselves and for their children (see the survey instrument in the electronic supplementary material for details).

Of the parents that said they would purchase the vaccine, about 13% indicated that they were uncertain about their purchase intentions either for themselves or for their child. Blumenschein et al. (2008) show that hypothetical bias can be reduced by treating respondents that state they are uncertain about purchase intentions as non-purchasers. Thus, parents with positive purchase intentions were defined as those that said that they were willing to pay for a vaccine and that said they “probably” or “definitely” would buy it if it were available.

Additionally, the parent is assumed to purchase the vaccine: (1) for herself only if she planned to continue purchasing it in the future and (2) for her child only if she believed that the child would continue to purchase vaccinations as an adult. This assumption is consistent with parents being told that the risk reductions assigned in the survey would materialize only for those who continue to receive the vaccinations annually through age 75. Then the decision to purchase the vaccine in the first period represents a continuing commitment to purchase the vaccine in the future, and the percentage risk reductions assigned represent the risk reductions resulting from this decision. Although baseline risk, age and other demographics may affect the choice, the percentage risk reductions are assigned independently of those factors.^17^

### Survey validity

The overall validity of the survey instrument might be assessed from three perspectives: (1) incentive compatibility and consequentiality of the stated preference question about intentions to purchase the vaccine, (2) practices followed in designing the survey instrument, and (3) whether data collected on intentions to purchase the vaccine are at least broadly consistent with rational behavior. As to the first point, the main finding of recent literature is that stated preference instruments may be consequential under certain conditions (Carson and Groves 2007 describe these conditions while Vossler et al. 2012 provide a detailed proof). While these conditions apply for the valuation of public goods, the situation is more challenging for private goods such as the vaccine described in this study.

These conditions, however, largely address the measurement of “total” value (willingness to pay for a good) rather than the marginal valuation of components of a good. In this study, the total value of the vaccine is of little interest; instead, attention is focused on the change in value for a change in risk reduction. Carson and Groves (2007) discuss the difficulty in attaining consequential private goods values because the desire to include the good in the choice set will likely lead to overstatement of the total willingness to pay. Nonetheless, Carson and Groves state that the valuation of an attribute (like risk reduction) in the private goods context can provide “useful estimates.”

Second, the survey approach adopted here includes many if not most of the elements outlined in the recent stated preference guidelines paper by Johnston et al. (2017). These guidelines include reminders of substitutes, pretesting and pilots, checking whether the purchase scenario is understood, and the use of debriefing questions to assess responses. Also, the uncertainty question about stated purchase intentions, while not completely endorsed in Johnston et al. (2017), avoids value cues and anchors because this question is asked after the valuation response.

Third, Table 2 reports tabulations showing the sensitivity of purchase intentions to changes in the price of the vaccine and the size of the risk reduction offered. Values in the table indicate the proportion of parents that say they would “probably” or “definitely” purchase a vaccine if it were available. At each of the five prices, for both parents and children, a greater fraction of parents indicated that they would purchase the vaccine at high levels of proportionate risk reduction than at low levels of risk reduction. All differences are statistically significant at the 5% level or less under one-tail tests for differences between proportions using independent samples. Additionally, at each value of proportionate risk reduction for both parents and children, the fraction of parents expressing an intention to purchase the vaccine tends to fall as price increases. Significant differences at 5% or lower are indicated in the table. These outcomes are consistent with econometric evidence presented in Table 3 to be discussed momentarily.

**Table 2:** Proportion of Parents That Would Buy Vaccine by Price and Risk Change (n = 2211)^a^

	Risk Reduction (%)	Proportion that would “probably” or “definitely” buy vaccine					
		$10	$20	$40	$80	$160	All
Parent	10	0.379	0.380	0.306^*^	0.258^**^	0.174^**^	0.300
	70	0.541	0.509	0.523	0.568	0.271^**^	0.482
	All	0.415	0.413	0.361	0.321	0.198	0.343
Child	20	0.392	0.335	0.336	0.232^**^	0.188^**^	0.294
	80	0.489	0.514	0.402^*^	0.452	0.289^**^	0.431
	All	0.442	0.420	0.370	0.330	0.236	0.360

^a^At each price, for both parents and children, the fraction of parents expressing intentions to purchase the vaccine is significantly greater at higher levels of proportionate risk reduction at 5% or lower under one-tail tests for differences between proportions using independent samples

^*^Indicates that for a given proportionate risk reduction, the fraction of parents expressing intentions to purchase the vaccine at the price shown is significantly different at 5% from the corresponding fraction at the price of $10 under a one-tail test for differences between proportions using independent samples. Significant differences at 1% indicated with ^**^

**Table 3:** Parents’ MWTP for Reductions in Heart Disease Risk. Bivariate Probit Estimates. n = 2211

	Proportionate Risk Reduction		Absolute Risk Reduction	
Covariate	Children	Parents	Children	Parents
Constant	−0.3729^**^ (0.0277)	−0.4195^**^ (0.0281)	−0.3725^**^ (0.0279)	−.4196^**^ (0.0282)
Proportionate Risk Reduction (*δ*_*i*_)	0.7404^**^ (0.0736)	0.9624^**^ (0.0865)	---^a^	---^a^
Absolute Risk Reduction (*ω*_*i*_)	---^a^	---^a^	0.0226^**^ (0.0022)	0.0227^**^ (0.0019)
Vaccine Price (*q*)	−0.0037^**^ (0.0005)	−0.0042^**^ (0.0005)	−0.0038^**^ (0.0005)	−0.0042^**^ (0.0005)
*ρ*_*ε*_	0.8912^**^ (0.0119)		0.8833^**^ (0.0120)	
Log-Likelihood	−2251.86		−2235.27	

^a^omitted variable

^**^denotes significance at the 1% level

## Econometric methods

This section begins by describing econometric methods used to estimate the relationship between MWTP for the vaccine and the proportionate reduction in heart disease risk that it offers. These methods build on those developed by Adamowicz et al. (2014) and are adapted later in this section to consider relationships between MWTP and absolute reductions in heart disease risks. The relationship between MWTP to reduce heart disease risk and the risk of this disease is developed from Eq. (12), in which MWTP for the vaccine is proportional to the reduction in heart disease risk that it offers.

12 $$ {W}_i={\gamma}_i{\Delta}_i\kern0.5em \left(i=p,k\right) $$

In this equation, *W*_*i*_ denotes the parent’s true (unobserved) willingness to pay (the parent index is suppressed to economize on notation) for the vaccine to reduce risks of heart disease for the parent (*p*) or the child (*k*), and, as discussed in Section 3, Δ_*i*_ denotes the experimentally assigned percentage reduction (expressed as a decimal fraction) in heart disease risk offered by the vaccine. Thus, *γ*_*i*_/100 is interpreted as the parent’s annual MWTP for a one percentage point reduction in heart disease risk.

Empirical methods applied build three additional features into Eq. (12). First, the possible discrepancy between true willingness to pay and stated (observed) willingness to pay $\left({\tilde{W}}_i\right)$ is modeled as a parent-specific random effect with non-zero mean (*α*_*i*_).^18^

13 $$ {\tilde{W}}_i={\alpha}_i+{W}_i+{\varepsilon}_i={\alpha}_i+{\gamma}_i{\Delta}_i+{\nu}_i $$

The constant *α*_*i*_ is included to reflect systematic tendencies to misstate true willingness to pay. The disturbance *ν*_*i*_ = *σ*_*i*_ *ε*_*i*_, assumed to be normally distributed with zero mean and variance of ${\sigma}_i^2$ (thus, *ε*_*i*_ has mean of zero and variance of unity), reflects the influence of household characteristics and unobserved heterogeneity among parents in determining stated willingness to pay for the vaccines.

Second, after subtracting the common parent/child vaccine price (*Q*) from both sides of Eq. (13), variables are mean-centered and the equation is re-parameterized by dividing through by *σ*_*i*_ as shown in Eq. (14).

14 $$ \left({\tilde{W}}_i-Q\right)/{\sigma}_i=\left({c}_i/{\sigma}_i\right)+\left({\gamma}_i/{\sigma}_i\right){\delta}_i-\left(1/{\sigma}_i\right)q+{\varepsilon}_i $$

Mean-centering eliminates the original constant term (*α*_*i*_/*σ*_*i*_). The new constant term (*c*_*i*_/*σ*_*i*_) is the mean of $\left({\tilde{W}}_i-Q\right)/{\sigma}_i$. Also, ${\delta}_i={\Delta}_i-{\overline{\Delta}}_i$ and $q=Q-\overline{Q}$.

Third, stated willingness to pay is latent: Parents only were asked whether they would be willing to pay the randomly assigned price *Q*. Thus, indicator variables are used to reflect the parent’s purchase decision (=1 if yes, implying that $\left({\tilde{W}}_i-Q\right)/{\sigma}_i\ge 0$; =0 if no, implying that $\left({\tilde{W}}_i-Q\right)/{\sigma}_i<0$).

Empirical estimates of Eq. (14) are obtained using bivariate probit to allow for the possibility that *ε*_*p*_ and *ε*_*k*_ may be correlated. Notice that the coefficient of *q* in Eq. (14) equals −1/*σ*_*i*_. This value can be used to recover the parent’s MWTP to reduce risk by one percentage point from the normalized coefficient *γ*_*i*_/*σ*_*i*_ (see Cameron and James 1987).^19^

Estimates of Eq. (14) minimize the potential for errors in inference arising from parental misstatement of purchase intentions in two ways. First, as previously discussed, parents expressing an intention to purchase the vaccine are treated as purchasers only if they also indicated that they would definitely or probably make this decision if the vaccine was actually available. Second, randomization of the percentage heart disease risk reductions and the vaccine price suggests that *δ*_*i*_ and *q* are distributed independently of the parent-specific characteristics included in the disturbance term (*ε*_*i*_). This feature supports consistent estimation of *γ*_*i*_ and *σ*_*i*_.

As mentioned previously, methods developed in this section can be adapted to yield estimates of the MWTP for absolute risk reductions in heart disease. One way to do this is to recover an estimate of the MWTP for a reduction in heart disease risk by 1 chance in 100 from estimates of Eq. (14) by dividing the estimate of *γ*_*i*_ by *R*_*i*_ where *R*_*i*_ denotes the level of (revised) perceived risk (expressed as chances in 100). An alternative approach is to begin the derivation of Eq. (14) by replacing Δ_*i*_ in Eq. (12) with Δ_*i*_ *R*_*i*_. This variable indicates the absolute reduction in heart disease risk (in chances in 100) that would be achieved by using the vaccine. The equation estimated then is Eq. (14) after replacing *δ*_*i*_ with mean-centered Δ_*i*_ *R*_*i*_, denoted as *ω*_*i*_. The coefficient of *ω*_*i*_ is interpreted as the MWTP for a 1 chance in 100 reduction in heart disease risk. Estimated coefficients of *ω*_*i*_ may not be consistent because this variable may be correlated with the disturbance. Nonetheless, these coefficients have the advantage that they do not need to be transformed in order to obtain values of MWTP for an absolute reduction in heart disease risk.

## Empirical results

This section presents empirical evidence on the relationship between MWTP to reduce risk of heart disease and baseline risk of this disease. The discussion is divided into four subsections. The first subsection contrasts two specifications in which: (1) MWTP for a proportionate change in heart disease risk is independent of baseline risk and (2) MWTP for an absolute change in baseline risk is independent of baseline risk. Estimates presented in the second subsection relax these restrictions. The third subsection considers how the relationship between MWTP and baseline risk is affected by changes in income and family size. The fourth subsection illustrates implications of the foregoing results for computing estimates of total morbidity benefits for a representative parent and child.

### Initial results

Columns 2 and 3 of Table 3 present results of bivariate probit estimates of Eq. (14), in which heart disease risk reduction is measured as a proportionate change. Estimates are based on the *n* = 2211 sample described in Section 3. By measuring heart disease risk reduction as a proportionate change, the restriction is imposed that MWTP for a given proportionate reduction in heart disease risk is independent of the level of baseline risk. An implication of this restriction is that the relationship between MWTP for an absolute reduction in risk (e.g., by 1 chance in 100) and baseline risk is negative and in the form of a rectangular hyperbola.

In the columns 2 and 3 estimates, the estimated correlation between disturbances is about 0.89, which differs significantly from zero at the 1% level and indicates that the use of bivariate probit yields an efficiency gain over separately estimating the parent and child equations using binomial probit. Additionally, in both child and parent equations: (1) estimates of the normalized coefficients of proportionate risk reduction (*γ*_*i*_/*σ*_*i*_) are positive and significantly different from zero at the 1% level and (2) estimates of coefficients of price are negative and significantly different from zero at the 1% level.^20^

Coefficients of the normalized proportionate risk reduction variables can be divided by 100 and then multiplied by the negative of the reciprocal of the respective coefficients of price to yield point estimates of the parents’ annual MWTP to reduce heart disease risk by one percentage point. The resulting un-normalized estimates, which do not depend on the level of baseline risk, are $2.29 in the case of parents’ own risk and $2.00 in the case of their children’s risk. Based on these estimates, the null hypothesis that parents’ MWTP for a one percentage point reduction in heart disease risk for themselves is equal to their MWTP for a one percentage point reduction in heart disease risk for their children is not rejected at conventional significance levels under a Wald test (*p*-value = 0.356).^21^

This outcome is consistent with optimizing behavior in the face of endogenous risks as discussed in Section 2, as well as with prior results of Dickie and Gerking (2007) and Adamowicz et al. (2014) in their studies of parents’ MWTP to reduce skin cancer and heart disease risks, respectively. Equality of parents’ MWTP for proportionate risk reductions to themselves and to their children also could be consistent with the exogenous risk model. Nonetheless, this model does not place restrictions on the relative magnitude of parents’ MWTP for morbidity risk reductions to themselves and to their children. Also, in the endogenous risk model, the estimated negative relationship between MWTP and baseline risk would suggest that the marginal utility of consumption is larger when healthy than when sick; whereas in the exogenous risk model this estimated relationship would suggest that the marginal utility of income is smaller when healthy than when sick.

The columns 2 and 3 regressions also imply that a representative parent’s MWTP to reduce heart disease risk to herself by 1 chance in 100 is $6.82 (= 2.29 x (100/33.59)) when evaluated at parents’ mean revised risk of $$ {\overline{R}}_p=33.59 $$ (see Table 1). For the representative parent’s child, the corresponding MWTP value would be $8.23 (= 2.00 x (100/24.30)) based on the mean of revised children’s risk of $$ {\overline{R}}_k $$ = 24.30. Thus, the representative parent perceives a lower level of heart disease risk for her child than for herself and is willing to pay more for a 1 chance in 100 reduction in risk to her child than she is for that same reduction in risk to herself. In prior studies (see Gerking and Dickie 2013), estimates of parents’ MWTP for absolute risk reductions for their children are mostly (but not uniformly) greater than parents’ MWTP for corresponding absolute risk reductions for themselves.

Columns 4 and 5 of Table 3 present results of estimating Eq. (14) subject to the restriction that MWTP for a reduction in heart disease risk by 1 chance in 100 is independent of baseline risk.^22^ The price variable is the same as that used in the columns 2 and 3 regressions, whereas heart disease risk reduction is measured by *ω*_*i*_, the mean-centered absolute risk change. All coefficient estimates in these equations are statistically different from zero at conventional levels. Un-normalized coefficient estimates suggest that at the margin parents are willing to pay $5.40 annually to reduce their own heart disease risk by 1 chance in 100 and $5.95 annually to reduce their children’s heart disease risk by this same absolute amount. These values are about 20–25% lower than those implied by the columns 2 and 3 regressions when evaluated at mean levels of heart disease risk for parents and their children.

The null hypothesis that parents’ MWTP to reduce their own heart disease risk by 1 chance in 100 is equal to their MWTP to reduce their children’s risk of heart disease by this same amount is not rejected under a Wald test (p-value = 0.458). The columns 4 and 5 estimates say little about the health state dependence of the marginal expected utility of income. In the case where MWTP to reduce baseline risk is constant in the face of changes in baseline risk, marginal expected utility of income when healthy may be greater than or less than marginal utility of income when sick in both the endogenous risk and exogenous risk models.

### Parents’ MWTP to reduce risk of heart disease and baseline risk: Further estimates

The relationship between MWTP for heart disease risk reduction and the baseline risk of this disease is further assessed by estimating alternative specifications to those shown in Table 3. Table 4 presents results of re-estimating regressions in Table 3, columns 2 and 3 to allow MWTP for a proportionate risk reduction to vary by risk level. Indicator variables for risk level are included as covariates in the parent and child equations and also are interacted with the proportionate risk reduction *δ*_*i*_ and the vaccine price *q*.^23^ All covariates are mean-centered. As previously indicated, proportionate risk reduction (*δ*_*i*_) and vaccine price (*q*) are randomly assigned, so their coefficients are consistently estimated. As demonstrated in the electronic supplementary material, coefficients of the interaction variables are consistently estimated as well. Coefficients of the indicator variables for revised perceived risk may not be consistently estimated in view of the probable non-zero correlation between these variables and the disturbance term. The possible inconsistency of these coefficients, however, is of little consequence because these constant term shifters play no role in subsequent analyses.

**Table 4:** Parents’ MWTP for Proportionate Reductions in Heart Disease Risk. Interactions with Baseline Risk. Bivariate Probit Estimates. n = 2211

Covariate	Children^a^	Parents^b^
Constant	−0.3794^**^ (0.0281)	−0.4289^**^ (0.0285)
Lowest Perceived Risk	−0.5244^**^ (0.0696)	−0.4136^**^ (0.0639)
Low Perceived Risk	−0.4311^**^ (0.0615)	−0.3075^**^ (0.0584)
High Perceived Risk	−0.3622^**^ (0.0635)	−0.1059 (0.0635)
Highest Perceived Risk	----c	----c
Proportionate Risk Reduction (*δ*_*i*_)	0.7500^**^ (0.0763)	0.9565^**^ (0.0894)
Vaccine Price (*q*)	−0.0037^**^ (0.0005)	−0.0041^**^ (0.0006)
Interaction of Proportionate Risk Reduction with Lowest Perceived Risk	−0.1214 (0.2202)	0.2108 (0.2414)
Interaction of Proportionate Risk Reduction with Low Perceived Risk	−0.0846 (0.1955)	0.2787 (0.2093)
Interaction of Proportionate Risk Reduction with High Perceived Risk	−0.1081 (0.1951)	0.2255 (0.2397)
Interaction of Vaccine Price with Lowest Perceived Risk	0.0017 (0.0013)	0.0003 (0.0013)
Interaction of Vaccine Price with Low Perceived Risk	−0.000001 (0.0011)	0.0008 (0.0011)
Interaction of Vaccine Price with High Perceived Risk	−0.0009 (0.0012)	−0.00006 (0.0012)
*ρ*_*ε*_	0.8983^**^ (0.0116)	
Log-Likelihood	−2199.88	

^**^denotes significance at the 1% level

^a^For children, lowest risk denotes revised risk between 0 and 14, low risk denotes revised risk between 15 and 20, high risk denotes revised risk between 21 and 30, highest risk denotes revised risk between 31 and 100

^b^For parents, lowest risk denotes revised risk between 0 and 20, low risk denotes revised risk between 21 and 30, high risk denotes revised risk between 31 and 46, highest risk denotes revised risk between 47 and 100

^c^denotes omitted variable

Estimates in Table 4 indicate that the coefficients of proportionate risk reduction (*δ*_*i*_) and vaccine price (*q*) differ significantly from zero at the 1% level in both the parent and child equations and are virtually identical numerically to those in columns 2 and 3 of Table 3. The estimated coefficients of the indicator variables for revised perceived risk suggest that parents perceiving relatively high risks of heart disease are more likely to express an intention to purchase the vaccine both for themselves and for their children. Individually, the estimated coefficients of the interaction variables of proportionate risk reduction with the indicator variables for revised perceived risk are not significantly different from zero at conventional levels. The same can be said for the coefficients of interactions variables of vaccine price with the indicator variables for revised perceived risk. These coefficients also do not jointly differ from zero under a likelihood ratio test with 12 degrees of freedom (p-value = 0.705). This outcome is consistent with the idea that: (1) parents’ MWTP for proportionate heart disease risk reductions for themselves and for their children are independent of changes in baseline risk and (2) MWTP for an absolute reduction in heart disease risk (e.g., by 1 chance in 100) is negatively related to baseline risk and in the form of a rectangular hyperbola.

Two alternative specifications of the regressions presented in Table 3, columns 4 and 5, make MWTP to reduce absolute risk: (1) a step-function of baseline risk and (2) a continuous piecewise linear function of baseline risk. Estimates are presented in the electronic supplementary material, Tables A4 and A5. In the step function estimates, covariates measuring absolute risk reduction (*ω*_*i*_) and price (*q*) are interacted with indicators of baseline risk. These indicators also are entered as constant term shifters. The pattern of estimated coefficients of interaction variables between *ω*_*i*_ and the level of perceived risk indicates that parents’ MWTP to reduce heart disease risk by 1 chance in 100 is highest at the lowest levels of risk for both parents and children. In the piecewise linear estimates, effects of absolute risk reduction also are nonlinear in baseline risk, with larger effects at lower risk. In both the step function and piecewise linear estimates, MWTP for risk reduction rises more gradually with declines in baseline risk than in the rectangular hyperbola estimates, particularly at the lowest risk levels.

Table 5 presents estimated values of MWTP for 1 chance in 100 risk reductions that are implied by the estimates in Tables 3 and 4 together with the step-function and piecewise linear estimates just described. Insignificant coefficients are disregarded in making these calculations. Estimates in columns 4 and 5 of Table 3 treat MWTP for a 1 chance in 100 risk of heart disease as a constant that does not depend on baseline risk. These estimates, $5.40 for parents and $5.95 for children, are reported in columns 2 and 6 of Table 5.

**Table 5:** Parents’ MWTP to Reduce Heart Disease Risk by 1 Chance in 100 by Perceived Risk Level. Alternative Methods

	Parents				Children			
Perceived Risk Level (chances in 100)	MWTP constant function of baseline risk	MWTP rectangular hyperbola in baseline risk	MWTP step function of baseline risk	MWTP piecewise linear function of baseline risk	MWTP constant function of baseline risk	MWTP rectangular hyperbola in baseline risk	MWTP step function of baseline risk	MWTP piecewise linear function of baseline risk
1	$5.40	$233.00	$14.20	$27.85	$5.95	$202.00	$14.07	$15.02
10	5.40	23.30	14.20	22.55	5.95	20.20	14.07	12.99
20	5.40	11.65	14.20	16.65	5.95	10.10	10.82	9.62
24.30	5.40	9.59	8.48	14.11	5.95	8.31	6.43	6.25
33.59	5.40	6.94	5.58	8.51	5.95	6.01	3.95	5.04
50	5.40	4.66	2.64	5.01	5.95	4.04	3.95	3.78
75	5.40	3.10	2.64	3.26	5.95	2.69	3.95	1.86
100	5.40	2.33	2.64	1.52	5.95	2.02	3.95	−0.07

Estimates in Table 4 are consistent with the notion that MWTP is a rectangular hyperbola in baseline risk. These estimates suggest (see columns 3 and 7 of Table 5) that when perceived risk is 100 (indicating a belief that a diagnosis of heart disease before age 75 is certain), parents’ MWTP to reduce their child’s risk of heart disease by 1 chance in 100 is $2.02 (i.e., the ratio of the estimated coefficient of *δ*_*i*_/100 to the negative of the estimated coefficient of *q*) and their MWTP to reduce their own risk of heart disease is $2.33. Estimates of MWTP at lower levels of risk are calculated by multiplying these values by 100/ *R*_*i*_. For instance, parents’ MWTP to reduce their own risk of heart disease by 1 chance in 100 evaluated at the mean of parents’ own perceived risk ($$ {\overline{R}}_p=33.59 $$, see Table 1) is $6.94. Parents’ estimated MWTP to reduce their children’s risk of heart disease evaluated at the mean of parents’ perceived risk for the child ($$ {\overline{R}}_k $$ = 24.30, see Table 1) is $8.31. Evaluating MWTP for 40% reductions in heart disease risk from means results in an increase in MWTP by nearly 70% for both parents and children. Additionally, a reduction in heart disease risk by 1 chance in 100 at *R*_*p*_ = *R*_*k*_ = 1 yields estimates for parents and children of $233.00 and $202.00, respectively.

At the lowest levels of perceived risk (*R*_*p*_ < 21, *R*_*k*_ < 15) in the step-function estimates, the representative parent’s MWTP for reductions in heart disease risk by 1 chance in 100 is $14.20 for themselves and $14.07 for their children (see columns 4 and 8 of Table 5). At means of perceived risk ($$ {\overline{R}}_p=33.59 $$ and $$ {\overline{R}}_k=24.30 $$), parents’ MWTP is $5.58 for themselves and $6.43 for their children. These estimates are close to those obtained by restricting MWTP for an absolute risk reduction to be independent of baseline risk (compare to columns 2 and 6 of Table 5). Differences are relatively small between MWTP values obtained from the step function and from the rectangular hyperbola when perceived risk exceeds 20. At the lowest values of perceived risk, however, differences between the two sets of estimates become relatively larger particularly as perceived risk levels approach *R*_*p*_ = *R*_*k*_ = 1. Evaluating MWTP for 40% reductions in heart disease risk from means results in an increase in MWTP by a factor of about 2.5 for parents and a factor of about 2.2 for children.

### MWTP, baseline risk, income, and family size

Discussion in Section 2 suggests that relationships between MWTP for heart disease risk reduction, baseline risk of heart disease, income, and number of children in a family also are of interest in comparing implications of the exogenous and endogenous risk formulations of the expected utility model. To check the role of income in determining MWTP and baseline risk the regressions in Table 3, columns 2 and 3 are re-estimated by adding four indicators for levels of total annual household income and interactions of these indicators with the proportionate risk reduction and price variables. As shown in the electronic supplementary material Table A6, estimates show that parents with higher income may be more likely to purchase the vaccine, but little tendency exists for MWTP for heart disease risk reduction to vary with income changes. This outcome is consistent with the endogenous risk model presented in Section 2 in which more of the risk reducing good is purchased as income increases.

The effect of number of children in a family on MWTP for proportionate reductions in risk of heart disease is investigated in the electronic supplementary material, Table A7, by adding to the regressions in Table 3, columns 2 and 3 covariates measuring the number of children present in the household together with interactions between number of children, proportionate risk reduction and vaccine price. Coefficients of the number of children in the household are negative and significantly different from zero at the 1% level and coefficients of interactions do not differ from zero at conventional levels. This outcome is consistent with discussion in Section 2: Including additional children in the endogenous risk model may have no effect on a parent’s MWTP to protect an individual family member, but would make it less likely that the vaccine would be purchased for one child when more children are present.

### Implications for estimating total morbidity benefits

As indicated in Section 1, the relationship between MWTP for risk reduction and baseline risk has implications for estimating total morbidity benefits. These implications can be illustrated using estimates previously presented in this section. Suppose that public policy could (somehow) reduce a representative parent’s heart disease risk from the mean level of $$ {\overline{R}}_p=33.59 $$ to a risk level of *R*_*p*_ = 20.^24^ How much would she be willing to pay for this risk reduction? If her MWTP to reduce risk by 1 chance in 100 is a constant that is independent of baseline risk, a possible answer could be obtained by multiplying the value in Table 5, column 2 by the number of units of risk reduction ($5.40 × 13.59 = $73.39).

In contrast, if her MWTP to reduce risk is inversely related to baseline risk, her total willingness to pay for the risk reduction may exceed this value. For instance, if her MWTP for risk reduction is represented by the Table 4 estimates (see also Table 5, column 3), her total willingness to pay would be $$ \$233\underset{20}{\overset{33.59}{\int }}\left(1/{R}_p\right){dR}_p=\$233\left[\ln (33.59)-\ln (20)\right]=\$120.80 $$. This value is about 60% larger than the value of $73.39 obtained using the Table 5, column 2 estimates.

Further comparisons show that: (1) Estimates of total willingness to pay developed from the step function estimates (Table 5, column 4) and the piecewise linear estimates (Table 5, column 5) also show that the assumption of independence between MWTP for risk reduction and baseline risk leads to an underestimation of total willingness to pay for heart disease risk reduction, (2) if a larger reduction in risk is considered ($$ {\overline{R}}_p=33.59 $$ to *R*_*p*_ = 10), then estimated total willingness to pay based on the rectangular hyperbola would be about 2.2 times greater than when based on the assumption that MWTP to reduce risk is independent of baseline risk ($282.30 vs. $127.39), and (3) estimates of total willingness to pay for reduced risk of heart disease for children follow similar patterns to those just described for parents.

## Conclusions

This paper theoretically and empirically examines the relationship between marginal willingness to pay (MWTP) to reduce morbidity risk from heart disease and baseline risk of this illness using unique data on parents’ perceptions of the risk of getting heart disease both for themselves and for their children. The main finding is that MWTP declines as baseline risk rises. A possible explanation for this result is that people engage in risk reducing efforts up to the point at which MWTP equates to marginal cost and marginal cost rises as more risk is eliminated. In any event, this outcome calls into question the standard practice in policy analysis of computing total benefits of health risk reduction based on the assumption that MWTP is independent of baseline risk. An example based on the empirical estimates shows that a 40% reduction in heart disease risk from the mean baseline risk for both parents and children results in a nearly 70% increase in MWTP.

Empirical results are supported by an expected utility model of parental decision-making in which health risk is treated either as exogenous or endogenous (i.e., influenced by parent behavior). Results suggest that parents are willing to pay an equal amount at the margin for equal absolute and proportionate risk reductions for themselves and their children. This outcome is consistent with optimizing behavior in the endogenous risk model, but may or may not be consistent with a similar model formulated with exogenous health risk. Empirical findings are thus consistent with the ideas that: (1) morbidity risk is endogenous, (2) marginal utility of consumption falls with the onset of illness (an outcome that may have additional implications for issues discussed elsewhere relating to insurance, life-cycle savings, and portfolio choice), and (3) parents adjust behavior to at least partly offset public policies aiming to reduce risk.

Results presented in this paper should be interpreted cautiously as additional research is needed to more conclusively establish the relationship between MWTP for health risk reduction and baseline risk. Future research might look at: (1) mortality rather than morbidity risk, (2) illnesses other than heart disease, (3) experimental methods, rather than stated preference methods, for estimating MWTP for health risk reduction, and (4) the shape of the relationship between MWTP and baseline risk at lower levels of risk than the present study is able to consider.

## Electronic supplementary material

ESM 1 (PDF 287 kb)

## Footnotes

1 Benefits of many types of morbidity effects of exposure to toxic substances have not been quantified and those that have been quantified are likely to be understated because they are often based on illness costs (see, for example, U.S. Environmental Protection Agency ( 2011), Tables 5.1 and 5.4).

2 Gerking and Dickie (2013) review estimates of parents’ MWTP to reduce health risks to themselves and their children.

3 In related models presented by Pratt and Zeckhauser (1996) and Liu and Neilson (2005, 2006), the bad health outcome is death, rather than illness.

4 A third health state, dead, could be added to the model (see Gerking et al. 2014) together with multiple time periods (see Dickie and Gerking 2007) to make clear that the parent might become ill and die at an earlier time than the child. These features, however, are not included in the model because they are not needed to support the empirical work conducted later on.

5 Exposure to fine particulates, for example, has been linked to heart disease (Brook et al. 2004).

6 With appropriate notational changes, the parent’s expected utility in equation (2) could alternatively be written so as to focus attention on the child.

7 For instance, further control of ambient ozone may reduce the productivity of staying indoors to cut the adverse effects of exposure to this pollutant.

8 If joint production is introduced in the endogenous risk model, as might occur if *X* both reduces risk and is a direct source of utility, utility terms generally cannot be eliminated from MWTP expressions.

9 This judgment would be altered, for example, if the parental provision of risk reduction to her children is subject to economies or diseconomies of scale.

10 A copy of the survey instrument is in the electronic supplementary material.

11 Information is unavailable for persons who either did not complete the screener or for persons who completed the screener, met the sample eligibility requirements, but did not complete the survey. Thus, it is not possible to compare characteristics of sample members to those of sample eligible persons.

12 Empirical results based on the *n* = 2211 subsample were checked against those obtained from the usable subsample of 1778 observations on unmatched parents. As shown in the electronic supplementary material, Table A1, results from the two subsamples are quite similar; however, estimates of MWTP for proportionate risk reductions were as much as 10%–15% larger in the unmatched parents subsample than in the augmented subsample of *n* = 2211 observations. Corresponding results for the *n* = 868 subsample of matched parents are presented in Adamowicz et al. (2014).

13 Another option would have been to exclude respondents who thought that Mr. A had the lesser chance of an accident on the grounds that they may have difficulty in understanding probability concepts. As it turns out, however, results reported in Section 5 are little changed whether these respondents are excluded or not: For instance, MWTP for a one percentage point reduction in risk derived from Table 3, columns 2 and 3 are about 3% higher if respondents answering the Mr. A question wrong are excluded (see the electronic supplementary material, Table A2 for details).

14 Based on data from the Framingham Heart Study (Lloyd-Jones et al. 1999), the average U.S. person faces a 27% risk of diagnosis with coronary artery disease before age 75. For females, this risk is 19% and for males it is 35%.

15 Focus group participants expressed only limited concerns about potential side effects of vaccines in general and about the heart disease vaccine in particular. In consequence, questions about possible respondent concerns about side effects of vaccines were not included in the survey.

16 Presenting the parent vaccine first resulted in a decrease in the probability that the vaccine would be purchased for the child, an effect that is significant at the 5% but not the 1% level. See Table A3 in the electronic supplementary material.

17 Additionally, Dickie and Gerking (2007) present a dynamic model of endogenous risk in a family setting. The model predicts that a parent’s MWTP to reduce risk equates to the marginal cost of risk reduction in the first period. In subsequent periods, individuals choose the amount of a risk-reducing good to equate the present value of the marginal cost of risk reduction across all periods. Thus, equations (10) and (11), used to distinguish between endogenous and exogenous risk models, also apply in a dynamic framework.

18 Thus, the specification applied (described momentarily), is based on the assumption that the randomly assigned risk changes and prices are unrelated to the difference between true and stated willingness to pay. This form of the model corresponds to theoretical findings in Carson and Groves (2007) and to empirical results in Taylor et al. (2010) demonstrating that for private goods, the difference between actual and hypothetical responses is captured in the intercept of the willingness to pay model.

19 If the risk reducing goods are direct sources of utility, a possibility suggested earlier, the correct cost value to use in equation (14) would be the randomly assigned cost variable presented to respondents net of monetized utility/disutility (*q* ^∗^ = *q* + *θ*). The term −(1/*σ*)*θ* that would be added to equation (14) can be treated as an additional component of the error already present in this equation. This term will affect the estimate of the constant term if it has a non-zero mean, but will not affect the point estimates of the coefficients of price and parent/child risk reduction because these variables were randomly assigned.

20 In a supplementary bivariate probit regressions, variables were entered to indicate whether the parent was first asked about purchasing the vaccine for herself or for her child. Results indicate that order of vaccine presentation was unimportant in the parent regression; however, vaccine purchases for the child were significantly (at the 5% level) lower when the parent vaccine was presented first. Nonetheless, estimated coefficients of proportionate risk reduction and price were virtually unchanged in both the parent and child equations in comparison with those presented in Table 3. This outcome would be expected because proportionate risk reduction, price, and order of vaccine presentation were randomly assigned.

21 The related null hypothesis that normalized coefficients of percent risk change and vaccine price are equal in the parent and child equations is not rejected at the 1% level under a Wald test (*p*-value = 0.021). This test, however, is stronger than that required to test for equality of parents’ MWTP for equal proportionate risk reductions for themselves and for their children.

22 In the survey, parents were presented with vaccines that offered proportionate reductions in heart disease risk and then were shown the corresponding absolute amount by which risk would be reduced using the risk scale.

23 The risk level indicators are defined at using the quartiles of the frequency distribution of parents’ perceived heart disease risk to themselves and to their children. In the parent equation, LOWEST denotes risk between 0 and 20, LOW denotes risk between 21 and 30, HIGH denotes risk between 31 and 46 and HIGHEST denotes risk greater than 46. In the child equation, LOWEST denotes risk between 0 and 14, LOW denotes risk between 15 and 20, HIGH denotes risk between 21 and 30, and HIGHEST denotes risk greater than 31.

24 For comparison, the U.S. government’s “Healthy People 2020” goals call for a reduction in *deaths* from coronary heart disease of about 20% relative to 2007 (from 129.1 to 103.4 deaths per 100,000 population) through better prevention, detection, and treatment of risk factors and other measures (https://www.healthypeople.gov/2020/topics-objectives/topic/heart-disease-and-stroke#two, last accessed: April 11, 2017).
