# Supplementary material for: Baseline risk and marginal willingness to pay for health risk reduction
Source: J Risk Uncertain. 2017 Dec 13;55(2):177–202. doi: 10.1007/s11166-017-9267-x (PMC5816778; doi:10.1007/s11166-017-9267-x)
Supplement: Supplementary file 1 — (PDF 287 kb) [file 11166_2017_9267_MOESM1_ESM.pdf]

Baseline Risk and Marginal Willingness to Pay for Health Risk Reduction

Electronic Supplementary Material

Shelby Gerking[a]
Wiktor Adamowicz[b]
Mark Dickie[c]
Marcella Veronesi[d]

[a] Department of Economics and Tilburg Sustainability Center, Tilburg University, P.O. Box 90153, 5000 LE Tilburg, the Netherlands, email: 56f100pu@gmail.com
[b] Department of Resource Economics and Environmental Sociology, University of Alberta, T6G 2H1, Edmonton, Alberta, Canada
[c] Department of Economics, University of Central Florida, P.O. Box 1400, Orlando, FL 32816-1400, United States
[d] Department of Economics, University of Verona, Via Cantarane 24, 37129, Verona, Italy; and Center for Development and Cooperation, ETH Zurich, Switzerland

TABLE OF CONTENTS

Supplementary figure	p. 3
Supplementary tables	p.4-p.10
Proof of consistency of Tables 3 and 4 estimates	p.11-p.13
Survey Instrument	p.14-p.59

Figure A1.

How many chances in 100 do you think you have of getting coronary artery disease before you reach age 75? Please mark the scale to show your answer.

1	11	21	31	41	51	61	71	81	91
2	12	22	32	42	52	62	72	82	92
3	13	23	33	43	53	63	73	83	93
4	14	24	34	44	54	64	74	84	94
5	15	25	35	45	55	65	75	85	95
6	16	26	36	46	56	66	76	86	96
7	17	27	37	47	57	67	77	87	97
8	18	28	38	48	58	68	78	88	98
9	19	29	39	49	59	69	79	89	99
10	20	30	40	50	60	70	80	90	100

Risk level: 36% chance of heart disease.

Table A1. Parents' MWTP for Reductions in Heart Disease Risk. Bivariate Probit Estimates. Unmatched parents. n = 1778.

	Proportionate Risk Reduction	
Covariate	Children	Parents
Constant	-0.4055** (0.0311)	-0.4616** (0.0315)
Proportionate Risk Reduction (δ_i)	0.7616** (0.0820)	1.0041** (0.0951)
Vaccine Price (q)	-0.0035** (0.0006)	-0.0038** (0.0006)
ρ_ε	0.9014** (0.01)	
Log-Likelihood	-1776.51	

[a] omitted variable

** denotes significance at the 1% level

Table A2. Parents' MWTP for Reductions in Heart Disease Risk. Bivariate Probit Estimates. Answered Mr. A Question Correctly. n = 1966.

	Proportionate Risk Reduction	
Covariate	Children	Parents
Constant	-0.3777** (0.0295)	-0.4245** (0.0299)
Proportionate Risk Reduction (δ_i)	0.7815** (0.0783)	1.0075** (0.0934)
Vaccine Price (q)	-0.0038** (0.0006)	-0.0043** (0.0006)
ρ_ε	0.8923** (0.0126)	
Log-Likelihood	-1994.30	

[a] omitted variable
** denotes significance at the 1% level

Table A3. Parents' MWTP for Reductions in Heart Disease Risk. Bivariate Probit Estimates. Check Whether Order of Vaccine Presentation Matters. n = 2211.

	Proportionate Risk Reduction	
Covariate	Children	Parents
Constant	-0.3730** (0.0278)	-0.4198** (0.0281)
Proportionate Risk Reduction (δ_i)	0.7380** (0.0735)	0.9631** (0.0867)
Vaccine Price (q)	-0.0037** (0.0005)	-0.0042** (0.0005)
Parent First[b]	-0.1294* (0.0553)	-0.0318 (0.0557)
ρ_ε	0.8921** (0.0119)	
Log-Likelihood	-2248.15	

[a] omitted variable
[b] parent vaccine offered first
** denotes significance at the 1% level
*denotes significance at 5% level

Table A4. Parents' Marginal Willingness to Pay for Absolute Reductions in Heart Disease Risk. Interactions with Baseline Risk. Bivariate Probit Estimates. n = 2211.

Covariate	Children[a]	Parents[b]
Constant	-0.2069** (0.0583)	-0.2708** (0.0494)
Lowest Perceived Risk	0.0542 (0.1468)	-0.0637 (0.0859)
Low Perceived Risk	-0.1259 (0.0723)	-0.1695** (0.0621)
High Perceived Risk	-0.2082** (0.0682)	-0.0654 (0.0666)
Highest Perceived Risk	---[c]	---[c]
Absolute Risk Reduction (ω_i)	0.0154** (0.0028)	0.0119** (0.0038)
Vaccine Price (q)	-0.0039** (0.0005)	-0.0045** (0.0006)
Interaction of Absolute Risk Reduction with Lowest Perceived Risk	0.0395** (0.0160)	0.0520** (0.0115)
Interaction of Absolute Risk Reduction with Low Perceived Risk	0.0268** (0.0080)	0.0263** (0.0059)
Interaction of Absolute Risk Reduction with High Perceived Risk	0.0097 (0.0057)	0.0132* (0.0054)
Interaction of Vaccine Price with Lowest Perceived Risk	0.0019 (0.0013)	0.0003 (0.0013)
Interaction of Vaccine Price with Low Perceived Risk	-0.00006 (0.0012)	0.0009 (0.0011)
Interaction of Vaccine Price with High Perceived Risk	-0.0009 (0.0012)	-0.00004 (0.0012)
ρ_ε	0.8934** (0.0112)	
Log-Likelihood	-2198.61	

**denotes significance at the 1% level * denotes significance at 5%

[a] For children, lowest risk denotes revised perceived risk between 0-14, low risk denotes revised perceived risk between 15-20, high risk denotes revised perceived risk between 21-30, highest risk denotes revised perceived risk between 31-100.

[b] For parents, lowest risk denotes revised perceived risk between 0-20, low risk denotes revised perceived risk between 21-30, high risk denotes revised risk between 31-46, highest risk denotes perceived revised risk between 47-100.

[c] denotes omitted variable

Table A5. Parents' Marginal Willingness to Pay for Absolute Reductions in Heart Disease Risk. Piecewise-Linear Interactions with Baseline Risk. Bivariate Probit Estimates. n = 2211.

Covariate	Children[a]	Parents[b]
Constant	-0.4636** (0.1548)	-0.5547** (0.1194)
Baseline Risk (lowest tercile)	-0.0236 (0.0174)	-0.0131 (0.0101)
Baseline Risk (middle tercile)	-0.0131 (0.0114)	0.0088 (0.0056)
Baseline Risk (highest tercile)	0.0068* (0.0030)	0.0021 (0.0025)
Absolute Risk Reduction (ω_i)	0.0450** (0.0165)	0.0289 (0.0158)
Vaccine Price (q)	-0.0046** (0.0017)	-0.0033* (0.0015)
Interaction of Absolute Risk Reduction with Baseline Risk (lowest tercile of baseline risk)	-0.0010 (0.0017)	-0.0020 (0.0014)
Interaction of Absolute Risk Reduction with Baseline Risk (middle tercile of baseline risk)	-0.0036** (0.0014)	-0.0020** (0.0006)
Interaction of Absolute Risk Reduction with Baseline Risk (highest tercile of baseline risk)	-0.0004** (0.0001)	-0.0002 (0.0001)
Interaction of Vaccine Price with Baseline Risk (lowest tercile of baseline risk)	-0.0002 (0.0001)	0.00003 (0.00009)
Interaction of Vaccine Price with Baseline Risk (middle tercile of baseline risk)	-0.0001 (0.0002)	-0.0001 (0.0001)
Interaction of Vaccine Price with Baseline Risk (highest tercile of baseline risk)	0.00003 (0.00005)	0.00003 (0.00004)
ρ_ε	0.8942** (0.0118)	
Log-Likelihood	-2197.83	

**Denotes significance at the 1% level. *Denotes significance at the 5% level

[a] For children, lowest tercile of baseline risk denotes revised perceived risk between 0-18, middle tercile denotes revised perceived risk between 18-25, highest tercile denotes revised perceived risk between 25-100.

[b] For parents, lowest tercile of baseline risk denotes revised perceived risk between 0-25, middle tercile denotes revised perceived risk between 25-38, highest tercile denotes revised perceived risk between 38-100.

Table A6. Marginal Willingness to Pay to Reduce Heart Disease Risk: The Role of Income. Proportionate Risk Reduction. Bivariate Probit Estimates. n=2211.

Covariate	Child Equation	Parent Equation
Constant	-0.5027** (0.0280)	-0.6534** (0.0283)
Proportionate Risk Reduction (δ_i)	0.5062* (0.2056)	0.5956** (0.2279)
Vaccine Price (q)	-0.0045** (0.0015)	-0.0041** (0.0015)
Lowest Income[a] (I_1)	---[b]	---[b]
Low Income[a] (I_2)	-0.0291 (0.0929)	0.1066 (0.9471)
High Income[a] (I_3)	0.1735 (0.0927)	0.2743** (0.0947)
Highest Income[a] (I_4)	0.2650** (0.0881)	0.3906** (0.0897)
$I_2 \cdot \delta_i$	0.3065 (0.2574)	0.5264 (0.2871)
$I_3 \cdot \delta_i$	0.1882 (0.2535)	0.2439 (0.2835)
$I_4 \cdot \delta_i$	0.3224 (0.2386)	0.5271 (0.2777)
$I_2 \cdot q$	0.0018 (0.0018)	0.0007 (0.0018)
$I_3 \cdot q$	0.0002 (0.0018)	-0.0008 (0.0018)
$I_4 \cdot q$	0.0007 (0.0017)	-0.0002 (0.0017)
ρ_ε	0.8912** (0.0121)	
Log-Likelihood	-2232.04	

.

[a] Total annual household income (I) is available by category. Indicators for this variable are defined according to whether this variable is: (1) < $50,000, (2) between $50,000 and $79,999, (3) between $80,000 and $124,999, or (4) $125,000 or more.

[b]Denotes omitted variable

** Denotes significance at the 1% level * Denotes significance at the 5% level

Table A7. Marginal Willingness to Pay to Reduce Heart Disease Risk: The Role of Number of Children. Proportionate Risk Reduction. Bivariate Probit Estimates. n=2211.

Covariate	Child Equation	Parent Equation
Constant	-0.3783** (0.0280)	-0.4236** (0.0283)
Proportionate Risk Reduction (δ_i)	0.7496** (0.0744)	0.9755** (0.0875)
Vaccine Price (q)	-0.0039** (0.0005)	-0.0043** (0.0006)
Number of Children (N)	-0.1126** (0.0277)	-0.0996** (0.0274)
$N \cdot \delta_i$	-0.0578 (0.0744)	-0.0690 (0.0737)
$N \cdot q$	-0.0009 (0.0005)	-0.0006 (0.0005)
ρ_ε	0.8900** (0.0120)	
Log-Likelihood	-2239.87	

.
[a] Denotes omitted variable
** Denotes significance at the 1% level

Consistency of Estimators Reported in
Tables 3 and 4

This appendix supports claims advanced in Sections 4 and 5 regarding consistency of certain estimates reported in Tables 3 and 4. The analysis utilizes the inessential simplification that Δ_i (see equation (12), Section 4) takes on values of one and zero to indicate high and low percentages of risk reduction. For expository purposes, drop the parent/child subscript from all covariates and include the household index h; thus Δ_h refers to the percentage risk reduction assigned to either the parent or child in household h, R_h refers to parent's revised perceived risk for either herself or her child in household h, and Q_h refers to the vaccine price assigned to household h. The variables δ_h, r_h, and q_h are the mean-centered counterparts of Δ_h, R_h, and Q_h respectively. In the demonstrations to follow, assume that the data are ordered so that the first n_1 observations have $\Delta_h = 1$ and the next n_2 observations have $\Delta_h = 0$ $(n_1 + n_2 = n)$. Estimates with risk reduction measured as chances in 100 presented in columns 4 and 5 of Table 3 are treated first, followed by estimates with risk reduction measured as a percentage presented in columns 2 and 3 of Tables 3 and 4.

As stated in Section 5, estimates in columns 4 and 5 of Table 3 may not be consistent. Although experimentally assigned values of vaccine price are distributed independently of the disturbance, values of ω_h (that is, mean-centered ΔR) remain correlated with ε_h as the sample size becomes arbitrarily large. More specifically,

$$\sum_{h=1}^{n} \omega_h \varepsilon_h = \sum_{h=1}^{n} (\Delta R)_h \varepsilon_h - m \sum_{h=1}^{n} \varepsilon_h = \sum_{h=1}^{n_1} R_h \varepsilon_h - m \sum_{h=1}^{n} \varepsilon_h \qquad \text{(A1)}$$

In equation (A1), m denotes the sample mean of $(\Delta R)_h$. If, as $n \to \infty$, $m \sum_{i=1}^{n} \varepsilon_h$ converges in probability to zero, then, given random assignment of Δ_h , $\sum_{h=1}^{n} \omega_h R_h$ converges in probability to the population covariance between R and ε. This covariance may not be equal to zero because in the endogenous risk model perceived risk is determined as an outcome of the utility maximizing process by the same set of factors that determine the value of the disturbance.

Hence, the coefficient of $(\Delta R)_h$ in the Table 3, columns 4 and 5 regressions may not be estimated consistently.

If the functional form applied is correct, then estimates in columns 2 and 3 of Tables 3 and 4 are consistent because the experimentally assigned values of proportionate risk change and vaccine price are distributed independently of the disturbance (ε_{ih}) (see Section 4, equation (14)).

Columns 2 and 3 of Table 4 adds the variables r_h^i, $\delta_h^i \cdot r_h^i$, and $p_h \cdot r_h^i$ to the covariates included in the corresponding columns of Table 3. The equation to be estimated is shown in equation (A2)

$$(\tilde{W}_h - Q_h)/\sigma = (c/\sigma) + (\gamma_1/\sigma)\delta_h - (1/\sigma)q_h + (\gamma_2/\sigma)r_h \\ + (\gamma_3/\sigma)\delta_h \cdot r_h + (\gamma_4/\sigma)q_h \cdot r_h + \varepsilon_h \qquad \text{(A2)}$$

Consider the covariance between $\delta_h \cdot r_h$ and r_h. If $\delta_h \cdot r_h$ has mean of μ, then this covariance is

$\sum_{h=1}^{n}(\delta_h \cdot r_h - \mu)\cdot r_h = \sum_{h=1}^{n}\delta_h \cdot r_h^2$, where

$$\sum_{h=1}^{n}\delta_h \cdot r_h^2 = \sum_{h=1}^{n}\Delta_h r_h^2 - \bar{\Delta}\sum_{h=1}^{n} r_h^2 = \sum_{h=1}^{n_1} r_h^2 - \frac{n_1}{n}\sum_{h=1}^{n} r_h^2 = \\ \left(n_1\sum_{h=1}^{n_1}\frac{1}{n_1}r_h^2\right) - \left(n_1\sum_{h=n_1+1}^{n}\frac{1}{n}r_h^2\right) = n_1\bar{r}_1^2 - n_1\bar{r}^2 \qquad \text{(A3)}$$

In equation (A3), $\bar{r}_1^2$ denotes the mean square of respondent provided perceived risk values in the subsample in which $\Delta_h = 1$ and $\bar{r}^2$ denotes the mean square of perceived risk values for the entire sample. Next, write $\bar{r}^2$ as

$$\bar{r}^2 = \frac{n_1}{n}\bar{r}_1^2 + \frac{n_2}{n}\bar{r}_2^2 \qquad \text{(A4)}$$

where $\bar{r}_2^2$ denotes the mean square of perceived risk values in the subsample in which $\Delta_h = 0$. In small samples, $\bar{r}_1^2$ and $\bar{r}_2^2$ will not in general be equal. Nonetheless, as n becomes arbitrarily large, given that Δ_h is experimentally assigned, $\bar{r}^2 = \bar{r}_1^2 = \bar{r}_2^2$ and in equation (A3), $\sum_{h=1}^{n}\delta_h \cdot r_h^2$ =0.

Thus, as n becomes arbitrarily large, $\delta_h \cdot r_h$ and r_h are uncorrelated. A similar argument establishes that as n becomes arbitrarily large, the variables δ_h, p_h, $\delta_h \cdot r_h$, and $p_h \cdot r_h$ are

mutually uncorrelated and uncorrelated with both r_h and ε_h^*. This outcome implies that estimates of coefficients in equations reported in Table 4, columns 2 and 3 are consistent except for the constant term and the coefficients of perceived risk level.

CONSENT **[DISPLAY]**

SURVEY INSTRUMENT

Title of Project: Family Heart Disease Risk and Prevention Survey.
Principal Investigator: Mark Dickie
Other Investigators: Shelby Gerking

You are being invited to participate in a research study. Whether you take part is up to you.

- The purpose of this research is to provide policy-makers with better information about what people believe about their own and their children's risks of getting heart disease later in life.
- You are invited to participate in a survey about heart disease prevention. If you agree to participate, you will be asked questions regarding your beliefs about risks of life-threatening illnesses, especially heart disease. If you participate you will also be asked about the value to you of heart disease prevention. The survey includes questions about you and about a child living with you.
- Your knowledge and opinions are important for this study. There is no right or wrong answer to the survey questions. If you participate, please just answer the questions as thoughtfully as you can.
- The survey takes about 25 minutes on average. Please take the survey only when you can give it your full attention and complete it in one sitting.

You must be 18 years of age or older to take part in this research.

Study Contact for questions about the study or to report a problem: If you have questions, concerns, or complaints: Dr. Mark Dickie, Department of Economics, University of Central Florida, Box 161400, Orlando, FL 32816-1400; 407-823-4730; mdickie@bus.ucf.edu. You may also contact Knowledge Networks at 800-782-6899.

IRB contact about your rights in the study or to report a complaint: Research at the University of Central Florida involving human participants is carried out under the oversight of the Institutional Review Board (UCF IRB). This research has been reviewed and approved by the IRB. For information about the rights of people who take part in research, please contact: Institutional Review Board, University of Central Florida, Office of Research and Commercialization, 12201 Research Parkway, Suite 501, Orlando, FL 32826-3246 or by telephone at 407-823-2901.

[DISPLAY]

The questions in the survey require more thoughtful consideration from you than some other surveys. Therefore, please complete the survey at a time when you can give it your full attention and when you can complete it in one sitting. We thank you in advance for your time and your careful attention to this survey.

[SP; TERMINATE IF SKIPPED]
I agree to give the survey my full attention and to complete it in one sitting.

[DISPLAY]
To get started we need to find out a little bit about you and the people living with you.

[RADIO]
[PROMPT IF SKIP]
Q0**.** Are you now married and living with your spouse?
Yes → Q0A
No → Q0bi
[RADIO]
[IF Q0=YES]
Q0a**.** How long have you been married to your current spouse?
Less than 1 year
1 to 5 years
6 to 10 years
11 to 15 years
16 to 20 years
More than 20 years

[RADIO]
[IF Q0=YES]
[IF SKIP, PROMPT WITH "YOUR ANSWER TO THIS QUESTION WAS NOT RECORDED. PLEASE ANSWER THE QUESTION IF YOU CAN."]
Q0b. Have you ever been married to anyone other than your current spouse?
Yes → Q0d
No → Q1

[RADIO]
[IF Q0=NO]
[PROMPT IF SKIP]
Q0bi. Do you now live with a partner?
Yes → Q0bii
No → Q0c

[RADIO]
[IF Q0BI=YES]
[IF SKIP, PROMPT WITH "YOUR ANSWER TO THIS QUESTION WAS NOT RECORDED. PLEASE ANSWER THE QUESTION IF YOU CAN."]

Q0bii**.** How long have you been living with your current partner?
Less than 1 year
1 to 5 years
6 to 10 years
11 to 15 years
16 to 20 years
More than 20 years
[CONTINUE WITH Q0C]

[RADIO]
[IF Q0=NO]
[IF SKIP, PROMPT WITH "YOUR ANSWER TO THIS QUESTION WAS NOT RECORDED. PLEASE ANSWER THE QUESTION IF YOU CAN."]

Q0c. Have you ever been married?
Yes → Q0d
No → Q1

[RADIO]
[IF Q0B=YES OR Q0C=YES]
[IF SKIP, PROMPT WITH "YOUR ANSWER TO THIS QUESTION WAS NOT RECORDED. PLEASE ANSWER THE QUESTION IF YOU CAN."]

Q0d. How many times have you been married, in all?
Once
Twice
Three times
Four or more times

[RADIO]
[IF Q0B=YES OR Q0C=YES]
[IF SKIP, PROMPT WITH "YOUR ANSWER TO THIS QUESTION WAS NOT RECORDED. PLEASE ANSWER THE QUESTION IF YOU CAN."]

[DISPLAY IF TERMINATE BASED ON ANY OF Q1 - Q5]
[DISABLE BACK BUTTON]
Unfortunately, you do not qualify for this survey. Thank you for your time.

[RADIO]
[PROMPT IF SKIP]
Q1. How many children now live with you in your household?
Answer options are 0,1,2,…10 or more
[IF Q1=0, GOTO TERMINATE SHOW]
[IF Q1=1 GOTO Q2]
[IF Q1>1, GOTO Q3]

[RADIO]
[IF Q1=1]
[PROMPT IF SKIP]
Q2. Is this child your biological child (your own "natural" child, <u>NOT</u> an adopted, step, or foster child)?
Yes
No
[IF Q2=NO, GOTO TERMINATE SHOW]
[IF Q2=YES, GOTO Q4]

[RADIO]
[IF Q1>1]
[PROMPT IF SKIP]
Q3. Of the [answer from Q1] children that live with you now, how many are your biological children (your own "natural" children, NOT counting adopted, step, or foster children)?
Answer options are 0,1,2,…10 or more
[IF Q3=0, GOTO TERMINATE SHOW]
[IF Q3=1, GOTO Q4]
[IF Q3>1, GOTO Q5]

[RADIO]
[PROMPT IF SKIP]
Q4. Is this child at least 6 years old, but younger than 17 years old?
Yes
No
[IF Q4=YES and (Q0=YES or Q0bi=YES), GO TO Q5A]
[IF Q4=YES and Q0=NO and Q0bi=NO, GO TO Q6]
[IF Q4=NO GoTo Terminate show]

[RADIO]
[IF Q3>1]
[PROMPT IF SKIP]
Q5. How many of these [answer from Q3] children are at least 6 years old, but younger than 17 years old?
Answer options are 0,1,2,…10 or more

[IF Q5=0, GOTO TERMINATE SHOW]
[IF Q5=1, AND (Q0=YES OR QOBI=YES), GOTO Q5A]
[IF Q5=1 AND Q0=NO AND Q0BI=NO, GO TO Q6]
[IF Q5>1, AND (Q0=YES OR Q0BI=YES), GOTO Q5B]
[IF Q5>1, AND Q0=NO AND Q0BI=NO, GOTO SELECTION OF SAMPLE CHILD BY BIRTHDAY ALT. 1]

[RADIO]
[IF (Q0=YES OR Q0BI=YES) AND EITHER Q4=1 OR Q5=1]
[PROMPT IF SKIP]
Q5A. Is this child also the biological child of the spouse(if q0=yes) / partner (if q0bi=yes) you currently live with?
Yes
No
[IF Q5A=YES, DOUBLE UP* AND GO TO DISPLAY BEFORE Q6]
[IF NO, GOTO Q6]

[*DOUBLE UP = IF THE SPOUSE OR PARTNER IS A PANELIST, HE/SHE GETS THE SAME SURVEY VERSION AS IS ASSIGNED TO THE RESPONDENT – SAME SETTINGS OF ATTRIBUTES IN CONJOINT.]

[DISPLAY IF Q5A=YES]
Your spouse **(IF Q0=YES) /** partner **(IF Q0BI=YES)** may also have the opportunity to take this survey. Although you might feel like discussing parts of the survey with your spouse **(IF Q0=YES) /** partner **(IF Q0BI=YES)** , please wait until after he or she has taken it before you talk about it. Thank you.

[RADIO]
[IF (Q0=YES OR Q0BI=YES) AND Q5>1]
[PROMPT IF SKIP]
[# OF OPTIONS = ANSWER TO Q5]
Q5B. Based on the answers you provided, you have [answer from Q5] biological children between the ages of 6 and 17 years who live with you. How many of these **[answer from Q5]** children are also the biological children of the spouse (if q0=yes) / partner (if q0bi=yes)you currently live with?
Answer options are 0,1,2,…10 or more
[IF Q5B=0, GOTO SELECTION OF SAMPLE CHILD BY BIRTHDAY ALT. 1]
[IF Q5B>0, DOUBLE UP]
[IF Q5B=1, GOTO Q6]
[IF Q5B>1, GOTO SELECTION OF SAMPLE CHILD BY BIRTHDAY ALT. 2]

[SELECTION OF SAMPLE CHILD BY BIRTHDAY ALT. 1]
[DISPLAY]
[IF Q5>1 AND (Q0=NO AND Q0BI=NO), OR IF Q5B=0]
Q5C. Based on the answers you provided, you have [answer from Q5] biological children between the ages of 6 and 17 years who live with you.

In the rest of this survey, we would like to ask questions about you and about <u>one</u> of these children – the child <u>whose birthday is coming up next</u>. When you are asked questions about your child, please only think of this child.

If two or more children happen to have the same birthday, please think of the [50%: youngest/ 50%: oldest] of them.
[GOTO Q6]
[SELECTION OF SAMPLE CHILD BY BIRTHDAY ALT. 2]
[DISPLAY]
[IF Q5B>1]
Q5D. Based on the answers you provided, you have [answer from Q5B] biological children between the ages of 6 and 17 years who live with you and who are also the biological children of the spouse (if q0=yes) / partner (if q0bi=yes)you currently live with.

In the rest of this survey, we would like to ask questions about you and about <u>one</u> of these children – the child <u>whose birthday is coming up next</u>. When you are asked questions about your child, please only think of this child.

If two or more children happen to have the same birthday, please think of the [50%: youngest/ 50%: oldest] of them.
[GOTO Q6]

[RADIO]
[PROMPT IF SKIP]
[Q6 ANSWER USED LATER IN SURVEY]
Q6. How old is this child?
6 years old
7 years old
8 years old
9 years old
10 years old

11 years old
12 years old
13 years old
14 years old
15 years old
16 years old

[DISPLAY]
In the remainder of this survey, we'll ask questions about you and about this child – your biological child aged **[answer from Q6]** years old who lives with you.

We ask you that you take your time in answering these survey questions and read carefully all the information given to you.

[GOTO Q7]

[PROMPT IF SKIP]
[RADIO]
[USE ANSWER TO Q7 FOR HE/SHE, HIS/HER DURING REST OF SURVEY.]
Q7. Is this child a boy or a girl?
Boy
Girl

[DISPLAY]
And now for a few questions about you….

[RADIO]
[PROMPT IF SKIP]
[ANSWER TO Q8B USED LATER IN SURVEY]
Q8b. What is your age?
Answer options are by individual year, from 18-55 inclusive.
[PROMPT IF SKIP]
[RADIO]
Q9. Are you a man or a woman?
Man
Woman

[Two additional questions to verify screener.]
[MP.]
[PROMPT IF SKIP]
[IF ANY ANSWER EXCEPT NONE, GO TO TERMINATE SHOW]
Q10. Has a doctor or other health care professional ever told you that you had any of the following conditions? Please check all that apply.

Coronary artery disease (this is sometimes called coronary heart disease)
Chest pain because of coronary artery disease (sometimes called angina)
Heart attack (a doctor might call this a myocardial infarction)
None of these conditions **[SP]**

[radio]
[PROMPT IF SKIP]
[IF q11 = YES, TERMINATE SHOW]
Q11. Have you ever had surgery to correct any condition caused by coronary artery disease? For instance, have you ever had a stent inserted in an artery, or have you had coronary bypass surgery?

1. Yes
2. No

[SAME RESPONSE FORMAT AS Q10, BUT Q12 NOT USED AS SCREENER.]
[IF SKIP, PROMPT WITH "YOUR ANSWER TO THIS QUESTION WAS NOT RECORDED. PLEASE ANSWER THE QUESTION IF YOU CAN."]

Q12. Now please think about your child's biological **<mother if Q9 = Man / father if Q9 = woman>. Has a doctor or health professional ever said that <he/she = opposite gender of respondent given in Q9>**
had any of the following conditions? Please check all that apply.
Coronary artery disease (this is sometimes called coronary heart disease)
Chest pain because of coronary artery disease (sometimes called angina)
Heart attack (a doctor might call this a myocardial infarction)
None of these conditions **[SP]**

[DISPLAY FOR "PROMPT IF SKIP"]:
You do not have to answer any question you do not wish to answer. But, we will not be able to proceed through the rest of the survey without your answer to this question.
[DISPLAY THE SKIPPED QUESTION BELOW THAT TEXT. IF RESPONDENT SKIPS AGAIN, THEN TERMINATE AND DISPLAY:]
Thank you for your time.

[IF SKIP, PROMPT WITH "YOUR ANSWER TO THIS QUESTION WAS NOT RECORDED. PLEASE ANSWER THE QUESTION IF YOU CAN."]
SD1. What is the highest level of schooling that you have completed?
Less than high school
High school graduate
GED or equivalent
Some college (including 2-year degree)
Graduate of 4-year college or university
Graduate or professional degree

[IF SKIP, PROMPT WITH "YOUR ANSWER TO THIS QUESTION WAS NOT RECORDED. PLEASE ANSWER THE QUESTION IF YOU CAN."]
SD2. Are you currently
Employed → SD2a
Not employed → SD4

[IF SD2=EMPLOYED]
[IF SKIP, PROMPT WITH "YOUR ANSWER TO THIS QUESTION WAS NOT RECORDED. PLEASE ANSWER THE QUESTION IF YOU CAN."]
SD3. How much total income do you earn <u>from your employment</u> annually?
$0
More than $0 to less than $5,000
$5,000 to less than $10,000
$10,000 to less than $20,000
$20,000 to less than $30,000
$30,000 to less than $40,000
$40,000 to less than $50,000
$50,000 to less than $60,000
$60,000 to less than $70,000
$70,000 to less than $80,000
$80,000 to less than $90,000
$90,000 to less than $100,000
$100,000 to less than $125,000
$125,000 to less than $150,000
$150,000 to less than $175,000
$175,000 to less than $200,000
$200,000 or more

[Multiple response format except last answer option]
[IF SKIP, PROMPT WITH "YOUR ANSWER TO THIS QUESTION WAS NOT RECORDED. PLEASE ANSWER THE QUESTION IF YOU CAN."]
SD4. Apart from earnings from employment, have you personally received any income from any other source during the past 12 months? Please check all other sources of income, such as

Unemployment compensation
Child support
Alimony
Dividends
Interest
Social Security
Welfare
Gifts

Any other income besides earnings from employment.

No other sources of income except earnings from employment

[IF SKIP, PROMPT WITH "YOUR ANSWER TO THIS QUESTION WAS NOT RECORDED. PLEASE ANSWER THE QUESTION IF YOU CAN."]

[validity check make sure "answer to SD6 >= answer to SD3"]
[If SD6<SD3, display "Your household's total income should be at least as large as your personal income." Then re-ask SD6.]

SD6. Please indicate the total annual income from <u>all sources</u> for all adults in your household. Please include all sources or income, including earnings from employment and any other income. Your <u>household's total annual income</u> is

$0
More than $0 to less than $5,000.
$5,000 to less than $10,000
$10,000 to less than $20,000
$20,000 to less than $30,000
$30,000 to less than $40,000
$40,000 to less than $50,000
$50,000 to less than $60,000
$60,000 to less than $70,000
$70,000 to less than $80,000
$80,000 to less than $90,000
$90,000 to less than $100,000
$100,000 to less than $125,000
$125,000 to less than $150,000
$150,000 to less than $175,000
$175,000 to less than $200,000
$200,000 or more

[radio]
[PROMPT IF SKIP]

W11. We would like to know how you feel about getting money now compared to getting money later. Please imagine that you have won a $100 prize. Suppose you were given the following options: You could either receive the $100 prize one month from now, or receive $LATER thirteen months from now. Which option would you choose? *Please select one response only.*

$100 one month from now
$LATER thirteen months from now

COMPUTE $LATER = K * $100, WHERE K IS RANDOMIZED OVER 1.05, 1.10, 1.20, 1.40, 1.80.

[DISPLAY]
Let’s now move to the main part of the survey which asks about risks to your health and to the health of your child. To help you pin down your answers, we want you to use a scale like the one you'll see after you click "Next".

[Display the following text above grid.. Below grid, text reads: Risk level __ %.] [Please delete the “Grid is 10x10” that occurs above all the scales.)

Here’s the scale. In a moment you will have the chance to use it, but first, notice that it has numbered squares beginning with 1 at the top left through 100 at the bottom right. When you are ready to move on, click the “Next” button below the scale.

> **{NOTES TO KN RE THE GRIDS: THE 100 SQUARES IN THE GRID SHOULD BE NUMBERED, BEGINNING WITH 1 IN THE UPPER LEFT HAND CORNER, TO 10 IN THE BOTTOM LEFT CORNER, 11 IN THE TOP OF THE SECOND COLUMN, AND SO ON DOWN AND ACROSS UNTIL 100 APPEARS IN THE SQUARE AT THE BOTTOM RIGHT HAND CORNER.**
>
> **THE INITIALLY DISPLAYED GRID SHOULD HAVE ALL 100 SQUARES COLORED BLUE. LATER, RED SQUARES ARE USED TO SHOW LEVELS OF RISK. THE TEXT BELOW THE GRID SHOULD INDICATE THE RISK LEVEL IN %. SO IF 10 OF 100 SQUARES ARE COLORED RED, THE RISK LEVEL IS 10%, ETC. ANY RED SQUARES SHOULD BE GROUPED IN CONSECUTIVELY NUMBERED SQUARES BEGINNING AT 1. SO IF 15 SQUARES ARE RED, THE RED ONES ARE SQUARES 1-15, AND THE TEXT BELOW THE GIRD READS 15% RISK.**
>
> **IN SOME CASES THE GRIDS ARE USED ONLY FOR DISPLAY AND SHOULD NOT ALLOW THE RESPONDENT TO CHANGE THE RISK LEVEL REPRESENTED BY THE NUMBER OF RED SQUARES. IN OTHER CASES, THE GRID SHOULD BE INTERACTIVE, SO THAT THE RESPONDENT CAN INDICATE A RISK LEVEL BY CLICKING A SQUARE IN THE GRID. FOR EXAMPLE, IF THE RESPONDENT SELECTS THE 56TH SQUARE, THEN ALL SQUARES FROM 1-56 SHOULD CHANGE FROM BLUE TO RED, AND THE RISK LEVEL SHOULD READ 56%. IF THE RESPONDENT THEN CLICKS ON SQUARE NUMBER 67, THEN SQUARES 57-67 ALSO BECOME RED, AND THE RISK LEVEL SHOULD READ 67%. IF THE RESPONDENT THEN CLICKS SQUARE 35, SQUARES 36-67 CHANGE BACK TO BLUE, AND ONLY SQUARES 1-35 ARE RED, WITH 35% DISPLAYED BELOW THE GRID. THE RESPONDENT RECORDS HIS/HER FINAL ANSWER BY CLICKING THE “NEXT” BUTTON.**
>
> **FINALLY, FOR SOME OF THE GRIDS, A THIRD COLOR BESIDES RED AND BLUE WILL BE NEEDED.}**
>
> **PLEASE LIGHTEN SLIGHTLY THE SHADE OF BLUE USED IN THE GRIDS SO THAT THE BLACK NUMBERS SHOW UP A LITTLE MORE CLEARLY. THANK YOU.**

[New screen display:]
Red squares in the scale show the chance that something will happen to make your health worse. For example, to show a 50% chance of worse health, half of the squares would be colored red. Remember that there are 100 squares in the scale, so a 50% chance is shown with 50 red squares:
[Static grid with 50 red, risk level of 50% indicated below grid.]

[New screen:] More red squares means a greater chance that your health will become worse. This scale shows a 75% chance, with 75 of the 100 squares colored red. The 75% chance of worse health also is shown numerically below the scale.
[Static grid with 75 red, risk level of 75% indicated.]

[New screen:] If something was 100% certain to make your health worse, all 100 squares would be red, as shown on the scale below. For a 0% chance, none of the squares would be red (they would all be blue).
[Static grid with 100 red squares, risk level of 100% indicated below grid.]

[NEW SCREEN:] This scale shows a 25% chance that your health will become worse. You can see that 25 squares are colored red. Chances of worse health also are shown numerically below the scale. **[Static grid with 25 red, 25% risk level.]**

[Display]
Now it's time for you to practice using the scale for a made-up example for Mr. A (he's not a real person), and his risk of having a car accident. Let's suppose that Mr. A's chances of being in a serious car accident are 33% or 33 in 100. You can use the scale to show this amount of risk by clicking on the number 33.
A1. Please click on number 33 in the scale now.

[DISPLAY Interactive grid with all 100 squares blue, and 0% risk level indicated below. Respondent should be able to click squares in the grid to show a risk level.]

[IF A1 answer = 33 (respondent clicks "Next" with risk level equal to 33, GoTo A3.]
[If respondent selects "Next" with the risk level <u>not</u> equal to 33:]
A2. *Oops! You must have clicked the wrong square in the scale. Please select the square numbered 33 in the scale below.*
[Interactive grid]
[IF A2 answer = 33, GoTo A3.]
[IF A2 answer NE 33, that is, if respondent selects "Next" with the risk level <u>not</u> equal to 33 for a second time, terminate.]

A3. Ms. B's chances of getting in a serious car accident are 1% or 1 in 100. Please show her risk by marking the scale below.
[Interactive grid with all blue squares initially and 0% risk level]

[IF A3 answer = 1, GOTO A5.]
[IF A3 answer NE 1, GOTO A4.]
[If respondent selects "Next" with the risk level **<u>not</u>** equal to 1:]
A4. *Oops! You must have clicked the wrong square in the scale. Please select the square numbered 1 in the scale below.*
[Interactive grid]
[IF A4 answer = 1, GoTo A5.]
[IF A4 answer NE 1, that is, if respondent selects "Next" with the risk level <u>not</u> equal to 1 for a second time, terminate.]

[RADIO]
A5. Which of these two people has the greater chance of being in an accident?
1. Mr. A
2. Ms. B

[If A5 answer=1, Display:] That's terrific. You might have thought that was too easy, but you would be surprised how many people get this wrong because they don't pay attention.
[THEN GOTO DISPLAY AFTER A6]

[If A5 answer NE 1:]
Are you sure? Remember, Mr. A's chances of getting in a wreck are 33 in 100, and Ms. B's chances are 1 in 100.

Let's have another look at the scales for these two people.

Mr. A's risk.
<DISPLAY Static grid with 33 red>

Ms. B's risk.
<DISPLAY Static grid with 1 red>

[Next screen] Remember Mr. A? He had a 33% chance of getting in a wreck. Ms B's chance was 1%.

[RADIO]
[IF A5 NE 1]
A6. Which of these two people has the greater chance of being in an accident?
1. Mr. A
2. Ms. B
[IF A6 ANSWER = 1, CONTINUE WITH DISPLAY BELOW]
[If A6 answer=2 OR SKIP, terminate.]

[Display]
In the rest of the survey, you'll have the chance to use the risk scale to estimate risks for yourself and for your child. Let's use the scale for two diseases that you or your child might get in the future. Let's do lung cancer first. Later on we'll ask about heart disease.

[RADIO]
[IF SKIP, PROMPT WITH "YOUR ANSWER TO THIS QUESTION WAS NOT RECORDED. PLEASE ANSWER THE QUESTION IF YOU CAN."]
S1. First, please think about a typical adult cigarette smoker. If you had to make an estimate, about how many packs of cigarettes do you think the average smoker smokes in a day?

1. Less than half a pack
2. About half a pack
3. About one pack
4. About one and half packs per day
5. About two packs per day
6. About two and half packs per day
7. About three packs per day
8. More than three packs per day
9. Don't know

S2. Think about a group of 100 average or typical smokers, who smoke cigarettes for all of their adult lives. How many smokers out of 100 do you think would get lung cancer?

Please mark your answer on the scale below. Remember, you can change your answer as often as you like until you click "Next."
[Interactive grid]
[Text below grid reads: [answer to S2] smokers out of 100 would get lung cancer.]

[display if the respondent does not select a square on S2:]
You did not indicate how many smokers out of 100 would get lung cancer.

S2a. Do you think that any smokers out of 100 would get lung cancer?
Yes → Send them back to S2.
No → Skip to S4

S3. Now please consider a group of 100 smokers who are diagnosed with lung cancer. Some smokers who get lung cancer live longer than five years, and others die within five years.

Out of 100 smokers who are diagnosed with lung cancer, how many do you think would die of lung cancer within five years of being diagnosed? Click the square that shows how many would die of lung cancer within five years of getting it.

[INTERACTIVE GRID WITH 100 BLUE SQUARES. WHEN RESPONDENT SELECTS A SQUARE, ALL THE SQUARES FROM 1 – THAT SQUARE RE-COLOR TO RED .
EXAMPLE: RESPONDENT ANSWERS S3 BY CLICKING SQUARE NUMBER 40. SQUARES 1-40 CHANGE TO RED. TEXT BELOW GRID HAS ONE LINE:

[ANSWER] SMOKERS OF 100 WITH LUNG CANCER WOULD DIE]

[display if the respondent does not select a square on S3:]

You did not indicate how many smokers would die lung cancer.

S3a. Do you think that any smokers out of 100 would die of lung cancer?
Yes → Send them back to S3.
No → Skip to S4

[RADIO]
[IF SKIP, PROMPT WITH "YOUR ANSWER TO THIS QUESTION WAS NOT RECORDED. PLEASE ANSWER THE QUESTION IF YOU CAN."]
S4. Have you ever smoked cigarettes?
1. Yes → S5
2. No → SKIP TO Display for heart disease after S10.

[RADIO]
[IF S4=YES]
[IF SKIP, PROMPT WITH "YOUR ANSWER TO THIS QUESTION WAS NOT RECORDED. PLEASE ANSWER THE QUESTION IF YOU CAN."]
S5. Have you smoked more than 100 cigarettes during your lifetime?
1. Yes →S6
2. No → display for heart disease after S10.

[RADIO]
[IF S5=YES]
[IF SKIP, PROMPT WITH "YOUR ANSWER TO THIS QUESTION WAS NOT RECORDED. PLEASE ANSWER THE QUESTION IF YOU CAN."]
S6. Have you smoked at least one cigarette per day during the past month?
1. Yes → S7
2. No → S8

[RADIO]
[IF S6=YES]
[IF SKIP, PROMPT WITH "YOUR ANSWER TO THIS QUESTION WAS NOT RECORDED. PLEASE ANSWER THE QUESTION IF YOU CAN."]
S7. About how many packs of cigarettes do you usually smoke in a day?

1. Less than half a pack
2. About half a pack
3. About one pack
4. About one and half packs per day
5. About two packs per day
6. About two and half packs per day
7. About three packs per day
8. More than three packs per day

GO TO S10.

[RADIO]
[IF S6=NO]

[IF SKIP, PROMPT WITH "YOUR ANSWER TO THIS QUESTION WAS NOT RECORDED. PLEASE ANSWER THE QUESTION IF YOU CAN."]
S8. Have you stopped smoking altogether?
1. Yes →S9
2. No → S10

[RADIO]
[IF S8=YES]
[IF SKIP, PROMPT WITH "YOUR ANSWER TO THIS QUESTION WAS NOT RECORDED. PLEASE ANSWER THE QUESTION IF YOU CAN."]
S9. How long ago did you stop smoking for the last time?
1. Less than 1 year ago
2. 1 to 5 years ago
3. 6 to 10 years ago
4. 11 to 15 years ago
5. 16 to 20 years ago
6. More than 20 years ago
CONTINUE WITH S10.

[ALL RESPONDENTS]
[DISPLAY]
Heart disease is the last disease that we'll ask you about. We'll focus on the most common form of heart disease, called *coronary artery disease*.

Coronary artery disease occurs when fatty deposits build up in the arteries that carry blood to the heart. The buildup of fatty deposits – called atherosclerosis – narrows the arteries and limits the flow of blood.

The buildup of fatty deposits starts in childhood, as explained in a recent article you may have seen in the *Wall Street Journal* (Nov 30, 2010, p D4).

[Display – new screen]
Coronary artery disease can cause chest pain and can lead to a heart attack. A heart attack occurs when one or more arteries are completely blocked with fatty deposits.

Heart disease is the leading cause of death in the United States.

In the rest of the survey, we'll use the terms "heart disease" and "coronary artery disease" to mean the same thing.

[RADIO]
[IF SKIP, PROMPT WITH "YOUR ANSWER TO THIS QUESTION WAS NOT RECORDED. PLEASE ANSWER THE QUESTION IF YOU CAN."]
H1. Have you ever heard or read about coronary artery disease, heart disease, or a heart attack?
1. Yes
2. No

[RADIO]
[IF SKIP, PROMPT WITH "YOUR ANSWER TO THIS QUESTION WAS NOT RECORDED. PLEASE ANSWER THE QUESTION IF YOU CAN."]
H2. Have you ever known anyone personally, like a friend or relative, who has been diagnosed with coronary artery disease or has had a heart attack?
1. Yes
2. No

[RADIO]
[IF SKIP, PROMPT WITH "YOUR ANSWER TO THIS QUESTION WAS NOT RECORDED. PLEASE ANSWER THE QUESTION IF YOU CAN."]
H4. Have you ever thought about the possibility that <u>you</u> might get coronary artery disease or have a heart attack?
1. Yes
2. No

[RADIO]

[DISPLAY]
Now, we'll ask you a few questions to help you estimate your own chances of getting coronary artery disease before you reach age 75. There are no right or wrong answers to these questions, please just make the most accurate estimate that you can.

[PROMPT IF SKIP]
H10. How many chances in 100 do you think you have of getting coronary artery disease before you reach age 75? Please mark the scale to show your answer.

[[Please change the grid for this question so that it starts with blue squares from 1-100, and allow respondents to select any square. Selecting a square recolors all squares up through the one selected to red.]

[Text below grid reads:] Risk level [answer to H10]% chance of heart disease.

[ANSWER TO H10 USED LATER IN SURVEY.]

[display if the respondent does not select a square on H10:]
[SHOW DISPLAY AND H10A ON THE SAME SCREEN]
You did not indicate any risk of getting coronary artery disease.

[PROMPT IF SKIP]
H10a. Everybody probably faces at least a small risk of getting heart disease Do you think that you have any chance at all of getting heart disease before age 75?
Yes → Send them back to H10.
No → go to H11.

[terminate if H10a is skipped]

[DISPLAY]
Now let's talk about your child's chances of getting heart disease before age 75. The questions about your child are similar to those we asked about you.

[radio]
[IF SKIP, PROMPT WITH "YOUR ANSWER TO THIS QUESTION WAS NOT RECORDED. PLEASE ANSWER THE QUESTION IF YOU CAN."]
H11. Have you ever thought about the possibility that your child might get coronary artery disease or have a heart attack sometime during **<his/her based on Q7>** life?
1. Yes

2. No

[Display]
Now please think about your child's chances of getting coronary artery disease before **[he/she based on Q7]** is age 75.

[PROMPT IF SKIP]
H15. How many chances in 100 do you think your child has of getting coronary artery disease before he reaches age 75? Please mark the scale below to show your answer.

[[Please change the grid for this question so that it starts with blue squares from 1-100, and allow respondents to select any square. Selecting a square re-colors all squares up through the one selected to red.]

[Text below grid reads:] Risk level: <answer to H15>%chance of heart disease.

[Answer to H15 used later in survey.]

[display if the respondent does not select a square on H15:]
[SHOW DISPLAY AND H15A ON THE SAME SCREEN]
You did not indicate any risk of getting a heart disease.

[PROMPT IF SKIP]

H15a. Everybody probably faces at least a small risk of getting heart disease Do you think that your child has any chance at all of getting coronary artery disease before age 75?
Yes → Send them back to H15.
No → continue.

[terminate if H15a is skipped]

[Display]

You may not be too sure about the risk estimates you just made. You'll be able to change these estimates later, after you've had a chance to review some information about heart disease.

Let's start with the average person's risk. According to medical research, the average person has about 27 chances in 100, or 27%, of getting coronary artery disease before reaching the age of 75. Click "Next" to see how the average person's

risk of heart disease compares to the estimates that you made for yourself and for your child.

[Fit three grid squares: parent risk scale (H10, show only the H10 answer as red, and remaining squares as blue), kid risk scale (H15 answer squares red, remaining blue, and 27% risk scale (27 red, 73 blue), all static.]

[Next screen display]
Of course, you and your child will probably not have the same risk as the average person, because chances of getting heart disease depend on six risk factors that are different for everyone.

[Next screen display]
Here are six important risk factors for heart disease.

[Display a checklist]
Heart Disease Risk Factors

- Gender
- Smoking
- Current health status
- Family history
- Exercise
- Diet

Let's briefly review each of these risk factors for you and your child.

[display centered] Gender

Heart disease risks are different for men and women. You can see how big the difference is by clicking "Next." [Splits the old gender slide into two slides to provide a better transition]

[DISPLAY]
NEXT SCREEN:

On average, heart disease risk is higher for males than for females.

[THEN SHOW TWO RISK SCALES SIDE-BY-SIDE, DISPLAY ONLY (RESPONDENTS CANNOT SELECT SQUARES IN GRID)].
[LEFT HAND SCALE SHOULD HAVE SQUARES 1- 19 COLORED RED, TEXT BELOW READS: AVERAGE WOMAN'S RISK: 19 %.]
[RIGHT HAND SCALE SHOULD HAVE SQUARES 1-35 COLORED RED, TEXT BELOW READS: AVERAGE MAN'S RISK: 35 %.]

[display centered] Smoking

Heart disease risks are different for smokers and nonsmokers. Click “Next” to see how big this difference is. Next screen:
[display]
Smokers face higher risks of coronary artery disease than non-smokers.

[Then display 2 risk scales side-by-side, display only.]
[Left hand scale should have squares 1-21 colored red. Text below reads: Average non-smoker’s risk: 21 %.]
[Right hand scale has squares 1-28 colored red. Text below reads: Average smoker’s risk: 28 %.]

Current Health Status

Now that we have considered gender and smoking status, let’s turn to your current health status and the current health status of your child.
[Display a checklist with Gender, Smoking checked off.]
If possible, please darken somewhat the checkmarks used in these checklists throughout the survey so that the checkmarks are more visible.
Heart Disease Risk Factors
- Gender
- Smoking
- Current health status
- Family history
- Exercise
- Diet

[RADIO]
[IF SKIP, PROMPT WITH “YOUR ANSWER TO THIS QUESTION WAS NOT RECORDED. PLEASE ANSWER THE QUESTION IF YOU CAN.”]
C1. Overall, would you say that your health is
1. Excellent
2. Very Good
3. Good
4. Fair
5. Poor

[RADIO]
[IF SKIP, PROMPT WITH “YOUR ANSWER TO THIS QUESTION WAS NOT RECORDED. PLEASE ANSWER THE QUESTION IF YOU CAN.”]
C2. How about your child’s health? Overall, would you say it is

1. Excellent
2. Very Good
3. Good
4. Fair

5. Poor

[IF Q9=1 (male) GO TO C3, IF Q9=2 (female) GO TO C3A]

[RADIO]
[IF SKIP, PROMPT WITH "YOUR ANSWER TO THIS QUESTION WAS NOT RECORDED. PLEASE ANSWER THE QUESTION IF YOU CAN."]
C3. Have you ever been told by a doctor or health professional that you need to do something (like take medication, stop smoking, change your diet or exercise more) to lower your blood pressure?
Yes
No
[Yes: GO TO C3B]
[No: Go to c4]

[RADIO]
[IF SKIP, PROMPT WITH "YOUR ANSWER TO THIS QUESTION WAS NOT RECORDED. PLEASE ANSWER THE QUESTION IF YOU CAN."]
C3A. Except during pregnancy, have you ever been told by a doctor or health professional that you need to do something (like take medication, stop smoking, change your diet or exercise more) to lower your blood pressure?
Yes
No
[Yes: GO TO C3B]
[No: Goto c4]

[RADIO]
[IF SKIP, PROMPT WITH "YOUR ANSWER TO THIS QUESTION WAS NOT RECORDED. PLEASE ANSWER THE QUESTION IF YOU CAN."]
C3B. [If Yes to C3 or C3A]: Are you currently taking medication for high blood pressure?
Yes
No
[continue with c4]

[RADIO]
[IF SKIP, PROMPT WITH "YOUR ANSWER TO THIS QUESTION WAS NOT RECORDED. PLEASE ANSWER THE QUESTION IF YOU CAN."]
C4. Has a doctor or health professional ever said that your child needs to do something (like take medication, change his/her Q7 diet, or exercise more) to lower his/her Q7 blood pressure?
Yes
No

[DISPLAY]
High blood pressure increases risk of coronary artery disease.

[THEN DISPLAY 2 RISK SCALES SIDE-BY-SIDE, DISPLAY ONLY.]

LEFT HAND SCALE SHOULD HAVE SQUARES 1-18 COLORED RED. TEXT BELOW READS: OPTIMAL BLOOD PRESSURE (LESS THAN 120/80): AVERAGE RISK IS 18%.
RIGHT HAND SCALE HAS SQUARES 1-43 COLORED RED. TEXT BELOW READS: VERY HIGH BLOOD PRESSURE (MORE THAN 160/100): AVERAGE RISK IS 43%.

[RADIO]
[IF SKIP, PROMPT WITH "YOUR ANSWER TO THIS QUESTION WAS NOT RECORDED. PLEASE ANSWER THE QUESTION IF YOU CAN."]
C5. Has a doctor or health professional ever told you that you need to do something (like take medication, change your diet, or exercise more) to lower your cholesterol?
Yes
No
[yes: goto c6 / no: go to c7]

[RADIO]
[IF SKIP, PROMPT WITH "YOUR ANSWER TO THIS QUESTION WAS NOT RECORDED. PLEASE ANSWER THE QUESTION IF YOU CAN."]
C6. [If C5=yes]: Are you currently taking any medication for high cholesterol?
Yes
No
[continue with c7]

[RADIO]
[IF SKIP, PROMPT WITH "YOUR ANSWER TO THIS QUESTION WAS NOT RECORDED. PLEASE ANSWER THE QUESTION IF YOU CAN."]
C7. Has a doctor or health professional ever said that your child needs to do something (like take medication, change his/her Q7 diet, or exercise more) to lower his/her Q7 cholesterol?
Yes
No

[Display] People with high cholesterol levels face higher risk of coronary artery disease, while people with normal cholesterol face lower risk.

[BELOW THE CURRENT TEXT, DISPLAY 2 RISK SCALES SIDE-BY-SIDE, DISPLAY ONLY.]
LEFT HAND SCALE SHOULD HAVE SQUARES 1-18 COLORED RED. TEXT BELOW READS: OPTIMAL TOTAL CHOLESTEROL (LESS THAN 180 MG/DL): AVERAGE RISK IS 18%.
RIGHT HAND SCALE HAS SQUARES 1-37 COLORED RED. TEXT BELOW READS: VERY HIGH TOTAL CHOLESTEROL (MORE THAN 240 MG/DL): AVERAGE RISK IS 37%.

[IF Q9=1 (male) GO TO C8, IF Q9=2 (female) GO TO C8A]

[RADIO]

[IF SKIP, PROMPT WITH "YOUR ANSWER TO THIS QUESTION WAS NOT RECORDED. PLEASE ANSWER THE QUESTION IF YOU CAN."]
C8. Has a doctor or health professional ever told you that you have diabetes?
Yes
No

[IF C8=YES GO TO C9, IF C8=NO GO TO C11]

[RADIO]
[IF SKIP, PROMPT WITH "YOUR ANSWER TO THIS QUESTION WAS NOT RECORDED. PLEASE ANSWER THE QUESTION IF YOU CAN."]
C8A. Except during pregnancy, has a doctor or health professional ever told you ever told you that you have diabetes?
Yes
No

[IF C8A=YES GO TO C9, IF C8A=NO GO TO C11]

[RADIO]
[IF SKIP, PROMPT WITH "YOUR ANSWER TO THIS QUESTION WAS NOT RECORDED. PLEASE ANSWER THE QUESTION IF YOU CAN."]
C9. How old were you when you were first told that you have diabetes?
10 years old or younger
11 to 20 years old
21 to 30 years old
31 to 40 years old
41 to 50 years old
51 to 55 years old

[RADIO]
[IF SKIP, PROMPT WITH "YOUR ANSWER TO THIS QUESTION WAS NOT RECORDED. PLEASE ANSWER THE QUESTION IF YOU CAN."]
C10. Are you currently taking medication for your diabetes?
Yes
No

[RADIO]
[IF SKIP, PROMPT WITH "YOUR ANSWER TO THIS QUESTION WAS NOT RECORDED. PLEASE ANSWER THE QUESTION IF YOU CAN."]
C11. Has a doctor or health professional ever said that your child has diabetes?
Yes
No

[IF C11=YES GO TO C12, IF C11=NO GO TO C13]

[RADIO]
[IF SKIP, PROMPT WITH "YOUR ANSWER TO THIS QUESTION WAS NOT RECORDED. PLEASE ANSWER THE QUESTION IF YOU CAN."]
C12. Is your child currently taking medication for **<his/her Q7>** diabetes?
Yes
No

[Display] People with diabetes are at much higher risk of coronary artery disease than people without this disease.

[DISPLAY]
People with diabetes face higher risk of coronary artery disease.
[THEN DISPLAY 2 RISK SCALES SIDE-BY-SIDE, DISPLAY ONLY.]
LEFT HAND SCALE SHOULD HAVE SQUARES 1-23 COLORED RED. TEXT BELOW READS: AVERAGE RISK WITHOUT DIABETES: 23%.
RIGHT HAND SCALE HAS SQUARES 1-62 COLORED RED. TEXT BELOW READS: AVERAGE RISK WITH DIABETES: 62 %.

[DISPLAY]
Weight in relation to height, called a "body mass index" or BMI, also is a risk factor for coronary artery disease. We'll calculate your body mass index and your child's body mass index in a moment. Please click "Next."

[RADIO]
[IF SKIP, PROMPT WITH "YOUR ANSWER TO THIS QUESTION WAS NOT RECORDED. PLEASE ANSWER THE QUESTION IF YOU CAN."]
C13. How tall are you? 1. Less than 4 feet 8 inches
2. 4 feet 8 inches to less than 4 feet 10 inches
3. 4 feet 10 inches to less than 5 feet 0 inches
4. 5 feet 0 inches to less than 5 feet 2 inches
5. 5 feet 2 inches to less than 5 feet 4 inches
6. 5 feet 4 inches to less than 5 feet 6 inches
7. 5 feet 6 inches to less than 5 feet 8 inches
8. 5 feet 8 inches to less than 5 feet 10 inches
9. 5 feet 10 inches to less than 6 feet 0 inches
10. 6 feet 0 inches to less than 6 feet 2 inches
11. 6 feet 2 inches to less than 6 feet 4 inches
12. 6 feet 4 inches to less than 6 feet 6 inches
13. 6 feet 6 inches to less than 6 feet 8 inches
14. 6 feet 8 inches or more

[RADIO]
[IF SKIP, PROMPT WITH "YOUR ANSWER TO THIS QUESTION WAS NOT RECORDED. PLEASE ANSWER THE QUESTION IF YOU CAN."]
C14. About how much do you weigh?

1. Less than 100 pounds

2. 100 to 109 pounds
3. 110 to 119 pounds
4. 120 to 129 pounds
5. 130 to 139 pounds
6. 140 to 149 pounds
7. 150 to 159 pounds
8. 160 to 169 pounds
9. 170 to 179 pounds
10. 180 to 189 pounds
11. 190 to 199 pounds
12. 200 to 209 pounds
13. 210 to 219 pounds
14. 220 to 229 pounds
15. 230 to 239 pounds
16. 240 to 249 pounds
17. 250 to 259 pounds
18. 260 to 269 pounds
19. 270 to 279 pounds
20. 280 to 289 pounds
21. 290 to 299 pounds
22. 300 or more pounds

[RADIO]
[IF SKIP, PROMPT WITH "YOUR ANSWER TO THIS QUESTION WAS NOT RECORDED. PLEASE ANSWER THE QUESTION IF YOU CAN."]
C15. How tall is your child?

1. Less than 2 feet 6 inches
2. 2 feet 6 inches to less than 2 feet 8 inches
3. 2 feet 8 inches to less than 2 feet 10 inches
4. 2 feet 10 inches to less than 3 feet 0 inches
5. 3 feet 0 inches to less than 3 feet 2 inches
6. 3 feet 2 inches to less than 3 feet 4 inches
7. 3 feet 4 inches to less than 3 feet 6 inches
8. 3 feet 6 inches to less than 3 feet 8 inches
9. 3 feet 8 inches to less than 3 feet 10 inches
10. 3 feet 10 inches to less than 4 feet 0 inches
11. 4 feet 0 inches to less than 4 feet 2 inches
12. 4 feet 2 inches to less than 4 feet 4 inches
13. 4 feet 4 inches to less than 4 feet 6 inches
14. 4 feet 6 inches to less than 4 feet 8 inches
15. 4 feet 8 inches to less than 4 feet 10 inches
16. 4 feet 10 inches to less than 5 feet 0 inches
17. 5 feet 0 inches to less than 5 feet 2 inches
18. 5 feet 2 inches to less than 5 feet 4 inches
19. 5 feet 4 inches to less than 5 feet 6 inches

20. 5 feet 6 inches to less than 5 feet 8 inches
21. 5 feet 8 inches to less than 5 feet 10 inches
22. 5 feet 10 inches to less than 6 feet 0 inches
23. 6 feet 0 inches to less than 6 feet 2 inches
24. 6 feet 2 inches to less than 6 feet 4 inches
25. 6 feet 4 inches to less than 6 feet 6 inches
26. 6 feet 6 inches to less than 6 feet 8 inches
27. 6 feet 8 inches or more

[RADIO]
[IF SKIP, PROMPT WITH "YOUR ANSWER TO THIS QUESTION WAS NOT RECORDED. PLEASE ANSWER THE QUESTION IF YOU CAN."]
C16. About how much does your child weigh?

1. Less than 20 pounds
2. 20 to 29 pounds
3. 30 to 39 pounds
4. 40 to 49 pounds
5. 50 to 59 pounds
6. 60 to 69 pounds
7. 70 to 79 pounds
8. 80 to 89 pounds
9. 90 to 99 pounds
10. 100 to 109 pounds
11. 110 to 119 pounds
12. 120 to 129 pounds
13. 130 to 139 pounds
14. 140 to 149 pounds
15. 150 to 159 pounds
16. 160 to 169 pounds
17. 170 to 179 pounds
18. 180 to 189 pounds
19. 190 to 199 pounds
20. 200 to 209 pounds
21. 210 to 219 pounds
22. 220 to 229 pounds
23. 230 to 239 pounds
24. 240 to 249 pounds
25. 250 to 259 pounds
26. 260 to 269 pounds
27. 270 to 279 pounds
28. 280 to 289 pounds
29. 290 to 299 pounds
30. 300 or more pounds

[DISPLAY] Based on your height and weight your Body Mass Index or BMI is approximately "COMPUTE.[insert a formula for BMI]". Although BMI is not a perfect

indicator, heart disease risks are higher for adults with BMI of 25 or above, and highest for adults with BMI 30 or above.

[PLEASE DISPLAY 3 GRIDS SIDE-BY-SIDE. LEFT HAND SCALE SHOWS 1-21 RED, TEXT BELOW SAYS BMI LESS THAN 25: AVERAGE RISK IS 21%.
MIDDLE SCALE SHOWS 1-24 RED, TEXT BELOW SAYS BMI BETWEEN 25 AND 30: AVERAGE RISK IS 24%.
RIGHT HAND SCALE SHOWS 1-32 RED, TEXT BELOW SAYS BMI OVER 30: AVERAGE RISK IS 32%.]

[DISPLAY] Based on your child's height and weight **<his/her Q7>** Body Mass Index or BMI is approximately "COMPUTE. [insert a formula for BMI]" For [boys/girls based on Q7] of age [answer to Q6] years old, heart disease risks are higher when BMI is [table lookup] or above, and highest when BMI is [table lookup] or above. But there is not enough data to tell *how* much higher the risk is for children.

[display centered] Family History

[DISPLAY]
The last three risk factors are family history, exercise and diet. We can't use the risk scales to tell you specifically *how much* these factors affect the average person's risk. But they are still important in determining whether a person will get coronary artery disease.

[Display a checklist with Gender, Smoking, and current health status checked off.]
Heart Disease Risk Factors
- Gender
- Smoking
- Current health status
- Family history
- Exercise
- Diet

Let's start with family history
[RADIO]
[IF SKIP, PROMPT WITH "YOUR ANSWER TO THIS QUESTION WAS NOT RECORDED. PLEASE ANSWER THE QUESTION IF YOU CAN."]
F1. Please think about your blood relatives on your side of your family. Have any of your blood relatives ever had a heart attack or been treated for coronary artery disease?
Yes
No
Don't Know

[RADIO]

[IF SKIP, PROMPT WITH “YOUR ANSWER TO THIS QUESTION WAS NOT RECORDED. PLEASE ANSWER THE QUESTION IF YOU CAN.”]

F3. Now please think about your child’s biological <mother’s if Q9 = Man / father’s if Q9 = woman> blood relatives. Have any of <her/his = opposite gender of respondent given in Q9> blood relatives ever had a heart attack or been treated for coronary artery disease?

Yes

No

Don’t know

[Display] Next, we will ask about exercise.

Exercise

[Display a checklist with Gender, Smoking, Family History, and Current Health status checked off.]

Heart Disease Risk Factors

- Gender
- Smoking
- Current health status
- Family history
- Exercise
- Diet

[RADIO]

[Display:] The American Heart Association recommends that adults in normal good health should get at least 5 hours weekly of moderate physical activity (such as brisk walking), or at least 1 hour weekly of vigorous activity (such as jogging) or some equivalent combination of moderate and vigorous activity.

[IF SKIP, PROMPT WITH “YOUR ANSWER TO THIS QUESTION WAS NOT RECORDED. PLEASE ANSWER THE QUESTION IF YOU CAN.”]

E1a. How much exercise do you get, compared to the American Heart Association recommendations?

Less exercise than recommended

About as much exercise as recommended

More exercise than recommended

[Display] The American Heart Association recommends that children in normal good health should participate in physical activity for 1 hour daily, including vigorous activity on at least 3 days per week.

[IF SKIP, PROMPT WITH “YOUR ANSWER TO THIS QUESTION WAS NOT RECORDED. PLEASE ANSWER THE QUESTION IF YOU CAN.”]

E2a. How much exercise does your child get, compared to the American Heart Association recommendations?

Less exercise than recommended

About as much exercise as recommended
More exercise than recommended

Diet

[Display]
The last item to cover on the list of heart disease risk factors is diet.
[Display a checklist with all items except Diet checked off.]
Heart Disease Risk Factors
    Gender
    Smoking
    Current health status
    Family history
    Exercise
    Diet

[RADIO]
[IF SKIP, PROMPT WITH "YOUR ANSWER TO THIS QUESTION WAS NOT RECORDED. PLEASE ANSWER THE QUESTION IF YOU CAN."]
D1. Would you say that you eat a healthy diet?
1. Very healthy
2. Somewhat healthy
3. Somewhat unhealthy
4. Very unhealthy

[RADIO]
[IF SKIP, PROMPT WITH "YOUR ANSWER TO THIS QUESTION WAS NOT RECORDED. PLEASE ANSWER THE QUESTION IF YOU CAN."]
D2. Would you say that your child eats a healthy diet?
1. Very healthy
2. Somewhat healthy
3. Somewhat unhealthy
4. Very unhealthy

[Display] The American Heart Association recommends that adults eat 4-5 cups of fruits and and vegetables daily.

[RADIO]
[IF SKIP, PROMPT WITH "YOUR ANSWER TO THIS QUESTION WAS NOT RECORDED. PLEASE ANSWER THE QUESTION IF YOU CAN."]
D2a. How much fruit and vegetables do you eat in a typical day?
Less than recommended
About as much as recommended
More than recommended

Next screen [please split the adult and child fruit/veg to two separate screens.]
[Display] The American Heart Association recommends that teenagers eat 4-5 cups of fruits and vegetables daily. Younger children should eat 2-4 cups of fruits/vegetables depending on their age and size.

[RADIO]
[IF SKIP, PROMPT WITH "YOUR ANSWER TO THIS QUESTION WAS NOT RECORDED. PLEASE ANSWER THE QUESTION IF YOU CAN."]
D3a. How much fruit and vegetables does your child eat in a typical day?
Less than recommended
About as much as recommended
More than recommended

[Display]
Now you have considered each of the main risk factors and you know how much they affect heart disease risk. If you have two or more risk factors, then your risk would be even higher.
People with several risk factors have the highest heart disease risk of all.

[New screen]
R1. Earlier, you said that your chances of getting heart disease before age 75 was <answer from H10>%. Now that you have thought about your risk factors, maybe you would like to change your answer. If so, use the risk scale below. If you do not wish to change your answer, just leave the scale marked as it is. When you are ready to move on click "Next."

[Interactive grid. When initially displayed, number of red squares = answer to H10, remaining squares are blue.]

R1a.
Suppose a doctor diagnoses you with coronary artery disease before age 75. What are the chances that you would die from coronary artery disease within five years of that diagnosis? Click the numbered red square that shows your chances of dying from heart disease within five years of being diagnosed.

[PLEASE MAKE THE GRID FOR THIS QUESTION HAVE 1-100 BLUE SQUARES AND ALLOW RESPONDENTS TO SELECT ANY SQUARE IN THE SCALE, CAUSING THE ALL SQUARES UP TO THE ONE SELECTED TO RE-COLOR AS RED.
TEXT BELOW SCALE SHOULD READ:]
Chance of dying from heart disease if diagnosed: <answer to R1a>%.

[display if the respondent does not select a square on R1a:]

[SHOW DISPLAY AND R1B ON THE SAME SCREEN]
You did not indicate any risk of dying from coronary artery disease.

[PROMPT IF SKIP]
R1b. Anyone diagnosed with heart disease probably faces at least a small risk of dying from the disease. Do you think that you have any chance at all of dying from heart disease before age 75?
Yes → Send them back to R1a.
No → go to R2.

[Display]
R2. Now let's continue with your child. Earlier, you said that your child's chance of getting heart disease before age 75 was **<answer from H15>%.**

If you would like to make a different estimate, please do so using the scale below. Then click "Next."

If you want to leave your estimate the same, just click "Next."

[Interactive grid. When initially displayed, number of red squares should equal answer to H15, remaining squares are blue]

R2a. Suppose a doctor has diagnosed your child with coronary artery disease before age 75. What do you think are the chances are that **<he/she Q7>** would die from coronary artery disease within five years of that diagnosis? Click the square that shows your child's chances of dying from heart disease within five years of being diagnosed.

[PLEASE MAKE THE GRID FOR THIS QUESTION HAVE 1-100 BLUE SQUARES AND ALLOW RESPONDENTS TO SELECT ANY SQUARE IN THE SCALE, CAUSING THE ALL SQUARES UP TO THE ONE SELECTED TO RE-COLOR AS RED.
TEXT BELOW SCALE SHOULD READ:]
Chance of dying from heart disease if diagnosed: <answer to R2a>%.

[display if the respondent does not select a square on R2a:]
[SHOW DISPLAY AND R2B ON THE SAME SCREEN]
You did not indicate any risk of dying from coronary artery disease.

[PROMPT IF SKIP]
R2b. Anyone diagnosed with heart disease probably faces at least a small risk of dying from the disease. Do you think that your child has any chance at all of dying from heart disease before age 75?

Yes → Send them back to R2a.
No → go to next section.

[Display]
Now that we've covered the information about risk factors, let's consider:

- How heart disease risks increase with age, and
- What the benefits are of reducing the risk of heart disease.

Heart disease risks over time

[Display]
[The following is just the copy of the previous client's word document titled "Displays following R2a --UCF Heart Disease Risk Survey"]

[DISPLAY THE NEXT TWO PARAGRAPHS ON THE SAME SCREEN]

You put your chances of getting coronary artery disease before you reach age 75 at [answer from R1] chances in 100. Your risk estimate means more than you might think at first. The next screen shows the risk you face between now and any age up through age 75.

(Please be patient as the next screen may take a moment to appear.)

[KN: The next screen displays a graph of cumulative risk by age. The instructions below explain the development of the graph for both the parent and the child.
The graph is built up from the function $G(a),$ where a is an index that runs from current age to 75:

$$G(a) = 100\left\{1 - \exp\left[\left(\frac{1-\exp\left(0.06\left(a-q\right)\right)}{1-\exp\left(0.06\left(75-q\right)\right)}\right)\ln(1-R)\right]\right\}.$$

In this equation, $q = \max(40,\ \text{Current Age}),$ and $R = \begin{cases} R1/100 \text{ for parent} \\ R2/100 \text{ for child} \end{cases}.$ Current age is Q8b for the parent, Q6 for the child.

The graph will show the positive quadrant of a standard (x, y) plane. The horizontal axis shows age in years from Current Age through 75. The vertical axis shows cumulative risk through each given age, in percent. So the range is (0,100), but most respondents will be far below 100% risk, so that the graph can be re-scaled accordingly.
The function to be graphed is as follows (please us a red curve to trace the function).
If Current Age ≥ 40, graph the function $F(a) = G(a),$ from above, over the domain (Current Age, 75).
If Current Age < 40, graph the function $F(a),$ defined by

$$F(a) = \begin{cases} 0 & \text{for Current Age } \leq a < 40 \\ G(a) & \text{for } 40 \leq a \leq 75 \end{cases}.$$

If the respondent points the mouse to the curve above an age a on the horizontal axis, please show a box should display "Your risk between now and age a is $F(a)\%.$" [Note the % sign added] for the parent, OR "Your child's risk between now and age a is $F(a)\%.$" [Again note % sign] when this is done for the child. For example, the respondent points to the graph above age 60, the box displays "Your risk between now and age 60 is <insert the value of the function *F(60)*>%." If the respondent subsequently points to the graph above a different age, the first pop-up disappears and a new one for the newly selected age is shown.

[Graph shows cumulative risk function for parent (respondent). The graph is labeled "Your Heart Disease Risk by Age"; the horizontal axis is labeled "Your Age"; the vertical axis is labeled "Your Risk".]

[Display]
Your heart disease risk profile is shown in the chart below. The height of the red curve shows your heart disease risk between now and any of the ages up to 75. To see how the chart works:

- Point to the red mark on the curve above the age of 75 to see your total risk of [answer to R1]% between now and age 75.
- Point to any red mark on the curve above any other age to see your risk between now and that age.

[Display] [KN: Note that the current version of the online survey has the wrong last sentence of the next paragraph.
Now let's continue with your child. You put your child's chances of getting coronary artery disease before <he/she Q7> reaches age 75 at [answer from R2] chances in 100. The next screen shows the risk your child faces between now and any age up through age 75.

[Graph shows cumulative risk function for child. The graph is labeled "Your Child's Heart Disease Risk by Age"; the horizontal axis is labeled "Your Child's Age"; the vertical axis is labeled "Your Child's Risk".]
[Display]
Your child's heart disease risk profile is shown below. The chart works much the same as the one for you. For instance,

- Point to the red mark on the curve above the age of 75 to see your child's total risk of [answer to R2]% between now and age 75.
- Point to any red mark on the curve above any age to see your child's age between now and that age.

- Notice that the chances of being diagnosed with heart disease before age 40 are practically zero.

Life with heart disease

[Display] You've thought a lot now about the risk of getting heart disease. But if you did get heart disease, what would your life be like? What are the benefits of reducing heart disease risk?

[RADIO]
L1. If you had heart disease, could it happen that you would have periodic episodes of chest pain or discomfort?
Yes
No

[Display] It often does happen that heart disease leads to chest pain or discomfort. While not all heart disease patients experience chest pain, it is the most common symptom of this disease.

By reducing your risk of heart disease, you increase your chances of living free from symptoms like chest pain.

[RADIO]
L2. If you had heart disease, could it happen that you would experience shortness of breath?
Yes
No

[Display] It often does happen that heart disease leads to shortness of breath. Heart disease patients are often limited in what they can do for this reason. Walking, climbing stairs, and other activities may seem more difficult than earlier in life.

By reducing your risk of heart disease, you would be better able to carry on your normal activities.

L2a. Can heart disease limit your ability to do household chores or to work in a job or business?
Yes
No

[display] Some heart patients have to rely on other people to take over some of their responsibilities at home or at work. Having to depend on others can be frustrating, costly, or can cause you to be less productive at work.

By reducing your risk of heart disease, you increase your chances of maintaining your independence.

[RADIO]
L3. If you had heart disease, do you think you might need more medical treatment, like more doctor visits and medication?

Yes
No

[Display] Medication is often prescribed for heart patients, and some people experience problems with side effects of medication. If it's a severe case of heart disease, you might need hospitalization and surgery, like a bypass operation.

By reducing your risk of heart disease, you increase your chances of living without lots of medication, medical treatment, or surgery.

[RADIO]

Reducing heart disease risks

[Display]
You may be interested in ways to reduce heart disease risk for you and your child. This part of the survey is about a program to reduce the risk of heart disease, and whether you would choose to participate.

The program is not yet available. We need your help in evaluating the program before it goes on the market.

[Display]
How the program works

- Each year, you would visit a doctor of your choosing to arrange for a blood test. The blood test will tell you and your doctor how much blockage of arteries is present.

- After each of the yearly blood tests, your doctor would give you one of several vaccines that would slow the build-up of fatty deposits in the arteries. The vaccine you get would be based on the outcome of the blood tests. The vaccine would be given by a shot in the arm.

- The vaccine would become available only after extensive testing shows that that it meets the same strict approval process used for other medications.

[Display]
Benefits and side effects

The vaccine would provide extra protection from heart disease over and above the benefits you can currently get from eating right and getting enough exercise. The

younger you are when you start the program, the greater the benefits. In a moment, we'll show you the benefits that you and your child would get.

But any medication can have side effects. In clinical trials, some people experienced side-effects like soreness in the arm, fatigue, or slight stomach upset. These side-effects generally disappeared within 1-2 days.

More serious side-effects very rarely occurred.
[Display]

What is the reduction in heart disease risk that you can expect from the vaccine?

First we will tell you about the risk reduction [version CA: insert "**YOUR CHILD" / VERSION AC: INSERT "YOU"**>], can expect from the vaccine, then we'll tell you about how much risk reduction [**VERSION CA**: insert "**YOU"/VERSION AC: INSERT "YOUR CHILD"]** can expect from the vaccine. We also will ask if you would be willing to pay your own money to get these benefits.

[RANDOMIZE QUESTION BLOCKS: VERSION CA: 50% WILL GET CHILD QUESTION BLOCK FOLLOWED BY ADULT QUESTION BLOCK; VERSION AC: 50% WILL GET CHILD QUESTION BLOCK FOLLOWED BY ADULT QUESTION BLOCK]

[START OF CHILD QUESTION BLOCK]
[Display]
Children who stay on the program from your child's age until age 75 would cut their risk by <H-KID>%.

RANDOMIZE H-KID = 20, 80.

To see what a **<H-KID>%** risk reduction would mean for your child, click "Next."
[DISPLAY]

Your child's risk reduction from the prevention program is shown in green. The risk your child would still face, if any, is shown in red.

[INSERT Risk Scale with boxes 1-HK colored red, and boxes (HK+1) through R2 colored green.]

[**COMPUTE DHK = (H-KID / 100) * (R2 ANSWER), AND if the result is not an integer, ROUND *UP*TO INTEGER. THEN COMPUTE HK = (R2 ANSWER) – DHK.]**

[DISPLAY]
To see how the prevention program would affect your child's risk by age, click "Next."
[DISPLAY]

The red curve shows your child's heart disease risk, without the prevention program. The green curve shows your child's heart disease risk if [he/she q7] stays on the program until age 75. Please use the cursor to point to different ages on both curves to show how much the prevention program will cut your child's risk. As you can see, the risk reduction starts small but gets bigger as your child gets older. That's why it is important to start young and stay on the program.

[Show the red and green curves as you already have them programmed.]

[Display]
We would like to know whether you would be willing to pay your own money to get these benefits for your child. If you have other children, you could put them in the program too. But for now please consider just the one child.

RANDOMIZE H_K OVER FIVE DOLLAR VALUES OF COST: 10, 20, 40, 80, 160.
[radio]
[PROMPT IF SKIP]

[New slide]. Would you be willing to pay $**< H_K > to put your child in the heart disease prevention program for the first year?**As you think over your answer, please consider two things:

•If your child was in the program, you would have less money available to pay for other family members to participate and to buy all the other things your family needs.

•If you put your child in the program for the first year, you may want to continue in future years to get the full heart disease prevention benefit. Of course, when your child becomes an adult it will be up to [him/her] to decide whether to participate.

So please take a moment to make sure your answer really reflects what you would do if this program were available.

To state your answer, please click "Next."

[sp; on the next screen]
W1. Would you be willing to pay **$< H_K > to put your child** in the heart disease vaccination program for the first year?
1. Yes
2. No

[If W1=1]
[sp]

W2a. You said that you would pay $**< H_K >** for your child to be in the heart disease prevention program for the first year. If the program was actually available, how certain are you that you would really do this?

1. Definitely
2. Probably
3.Uncertain

[MP; IF W1=1]

W3a. Which of the following reasons best describes why you would put your child in the program? (Please mark all that apply.)

1. The risk reduction is worth the expense.
2. It's important to start young to reduce heart disease risk.
4. I would spend whatever it takes to reduce my child's heart disease risk.
5. The program is better than other ways of reducing heart disease risk.
6. Some other reason.

[GO TO W4]

[IF W1=2]
[SP]
W4a. Which of the following reasons best describes why you would not put your child in the program? (Please mark all that apply.)

1. The risk reduction is too far in the future to justify the expense.
2. My child might not stay on the program as an adult, so there is no sense paying for it now.
3. There are other ways to spend money, including on health, that are better than this program.
4. My child can reduce heart disease risks without the program.
5. I don't believe that the prevention program would really work as described.
6. The program is too expensive.
7. I'm not that worried about my child's heart disease risk.
8. I already do enough to protect my child against heart disease.
9. I cannot afford the program
10. Some other reason.

[radio]
[if q0=yes or if q0bi=yes]
[PROMPT IF SKIP]

W4. Do you believe that your spouse (if q0=yes) / partner (if q0bi=yes)would agree with your decision about whether or not to enroll your child in the program?

Yes
No
[END OF CHILD QUESTION BLOCK]

[start of adult question block]
[Display]
Adults who stay on the program from your age until age 75 would cut their heart disease risk by **<H-PAR>**%.

RANDOMIZE H-PAR = 10, 70.

KEEP H-KID > H-PAR. Of the 4 possible combinations of (20,80) x (10,70), only 3 should actually be administered. The (H-KID=20, H-PAR=70) pair should not be used.

To see what a **<H-PAR>**% risk reduction would mean for you, click "Next."

COMPUTE DHP = (H-PAR / 100) * (R1 ANSWER), AND if result is not an integer, ROUND *UP*TO INTEGER. THEN COMPUTE HP = (R1 ANSWER) – DHP.

[DISPLAY]

Your risk reduction from the prevention program is shown in green. The risk you would still face, if any, is shown in red.

[INSERT Risk Scale with boxes 1-HP colored red, and boxes (HP+1) through R1 colored green.]

[DISPLAY]

To see how the program would affect your risk by age between any age and age 75, click "Next."

[DISPLAY]
The red curve shows your heart disease risk, without the prevention program. The green curve shows your heart disease risk if you stay on the program until age 75. Use the cursor to point to different ages on the two curves to find out how much the program will cut your heart disease risk. As you can see, the risk reduction starts small but gets bigger as you get older. That's why it is important to start now and stay on the program.

[Show the red and green curves as you already have them programmed.]

[Display]

We would like to know whether you would be willing to pay your own money to get these benefits.

Please use the same dollar price for H_P as was used for H_K in question W1 above.

[radio]
[PROMPT IF SKIP]

[DISPLAY]
WOULD YOU BE WILLING TO PAY $<H_P> TO PARTICIPATE IN THE HEART DISEASE VACCINATION PROGRAM FOR THE FIRST YEAR?

As you think over your answer, please consider two things:

- If you are in the program, you would have less money available to pay for other family members to participate and to buy all the other things your family needs.

- If you are in the heart disease vaccination program for the first year, you may want to continue in future years to get the full heart disease prevention benefit.

So please take a moment to make sure your answer really reflects what you would do if this program were available.

To state your answer, please click “Next.”

W5a. Would you be willing to pay $**< H_P >** to participate in the heart disease vaccination program for the first year?
Yes
No

[If W5=Yes Go to W6. If W5=NO go to W8]

W6a. You said that you would pay $<H_P> to participate in the heart disease prevention program for the first year. If the program was actually available, how certain are you that you would really do this?

1. Definitely
2. Probably
3. Uncertain

[If W6=1,2,3 Go to W7]

W7. Which of the following reasons best describes why you would choose to participate in the heart disease prevention program? (Please mark all that apply.)

1. The risk reduction is worth the expense.
2. It's important to start young to reduce heart disease risk.
3. If I develop good health habits now, it's likely the habits will continue in the future.
4. I would spend whatever it takes to reduce my heart disease risk.
5. The program is better than other ways of reducing heart disease risk.
6. Some other reason.

[GO TO END OF ADULT QUESTION BLOCK]

W8. Which of the following reasons best describes why you would not participate in the heart disease prevention program? (Please mark all that apply.)

1. The risk reduction is too far in the future to justify the expense.
2. There are other ways to spend money, including on health, that are better than this program.
3. I can reduce heart disease risks without the program.
4. I don't believe that the prevention program would really work as described.
5. The program is too expensive.
6. I'm not that worried about my heart disease risk.
7. I already do enough to protect my child against heart disease.
8. I cannot afford the program.
9. Some other reason.

[END OF ADULT QUESTION BLOCK]

[radio]

[IF q0=no AND q0bi=no, survey ends here]

[IF q0=yes or q0bi=yes]

We have one more question about your child's possible participation in the heart disease prevention program.

To get the full risk reduction of the prevention program, your child would have to stay on the program for many years. During that time, your family's financial situation could change in unexpected ways.

We would like to find out whether your decision would be affected if your family's financial situation changed.

[SHOW THE FOLLOWING DISPLAYS AND W9 If W2a =1 or 2] but only [IF q0=yes or q0bi=yes]

Suppose that you personally had a new expense. For example, suppose that you felt obligated to give financial help to a relative on your side of the family, or that you had an expensive medical procedure, or that you lost money on an investment that you personally had made. Suppose that the total cost to you is $X per year, for the next year.

At the same time, suppose that your spouse (if q0=yes) / partner (if q0bi=yes) unexpectedly received an extra $Y of income per year for the next year, from some source.

[Please randomize $X so that 50% of respondents get X = 2% of lower limit of answer to SD6, and 50% get X = 10% of lower limit to answer to SD6, given that lower limit is at least $5000. If the lower limit is less than $5000, set $X =$500.] Please randomize $Y so that 50% get Y=0.5X and 50% get Y=1.5X.
[Display]
Now please consider whether these changes in your family's finances would affect whether you would enroll your child in the heart disease prevention program.

[Display]

[radio]
[PROMPT IF SKIP]

W9. If you had extra expenses of $X per year and your spouse (q0=yes) / partner (q0bi=yes) had extra income of $Y per year, for the next year, would you be willing to pay $**< H_K >** for your child to enroll in the prevention program for the first year?
Yes
No

[If Q0=yes or q0bi=yes]
[SHOW THE FOLLOWING DISPLAYS AND W10 If W1=NO or ifW2a =3]

Suppose that your spouse (if q0=yes) / partner (if q0bi=yes) personally had a new expense. For example, suppose that **<he/she>** felt obligated to give financial help to a relative on <**his/her**> side of the family, or that <**he/she>** had an expensive medical procedure, or that <**he/she**> lost money on an investment that <**he/she**> personally had

made. Suppose that the total cost to your spouse (if q0=yes) / partner (if q0bi=yes) is $X per year, for the next year.

At the same time, suppose that you unexpectedly received an extra $Y of income per year for the next year, from some source.

[IF Q9=MAN, USE SHE/HER IN PREVIOUS PARAGRAPH. IF Q9=WOMAN, USE HE/HIS.]

[Please randomize $X so that 50% of respondents get X = 2% of lower limit of answer to SD6, and 50% get X = 10% of lower limit to answer to SD6, given that lower limit is at least $5000. If the lower limit is less than $5000, set $X = $500.] Please randomize $Y so that 50% get Y=0.5X and 50% get Y=1.5X.

[Display]
Now please consider whether these changes in your family's finances would affect whether you would enroll your child in the heart disease prevention program.

[radio]
[PROMPT IF SKIP]

W10. If your spouse (q0=yes) / partner (q0bi=yes) had extra expenses of $X per year and you had extra income of $Y per year, for the next year, would you be willing to pay $< **H_K** > every year for your child to enroll in the prevention program?
Yes
No

Next screen

[IF Q0=NO AND Q0BI=NO, SURVEY IS FINISHED]
[if Q0=yes or Qobi=YES, show sd7a or B and sd9]

We have just two more questions.

[RADIO]
[IF Q0=YES]
SD7a. Who takes primary responsibility for making health care decisions for your child?
You
Your spouse
You and your spouse jointly
Someone else

[RADIO]
[IF Q0=NO AND Q0BI=YES]
SD7b. Who takes primary responsibility for making health care decisions for your child?
You
Your partner
You and your partner jointly
Someone else

[NUMBER BOX]
SD9. What is the largest amount of money that you would be willing to spend on yourself during one month, without consulting your spouse (if q0=yes) / partner (if q0bi=yes)?
